# Influence of angiotensin II on the gut microbiome: modest effects in comparison to experimental factors

**DOI:** 10.1093/cvr/cvae062

**Published:** 2024-03-22

**Authors:** Rikeish R Muralitharan, Michael E Nakai, Matthew Snelson, Tenghao Zheng, Evany Dinakis, Liang Xie, Hamdi Jama, Madeleine Paterson, Waled Shihata, Flavia Wassef, Antony Vinh, Grant R Drummond, David M Kaye, Charles R Mackay, Francine Z Marques

**Affiliations:** Hypertension Research Laboratory, School of Biological Sciences, Faculty of Science, Monash University, 18 Innovation Walk, Clayton, 3800 Melbourne, Australia; Institute for Medical Research, Ministry of Health Malaysia, Kuala Lumpur, Malaysia; Victorian Heart Institute, Monash University, 631 Blackburn Road, Clayton, 3800 Melbourne, Australia; Hypertension Research Laboratory, School of Biological Sciences, Faculty of Science, Monash University, 18 Innovation Walk, Clayton, 3800 Melbourne, Australia; Hypertension Research Laboratory, School of Biological Sciences, Faculty of Science, Monash University, 18 Innovation Walk, Clayton, 3800 Melbourne, Australia; Victorian Heart Institute, Monash University, 631 Blackburn Road, Clayton, 3800 Melbourne, Australia; Hypertension Research Laboratory, School of Biological Sciences, Faculty of Science, Monash University, 18 Innovation Walk, Clayton, 3800 Melbourne, Australia; Hypertension Research Laboratory, School of Biological Sciences, Faculty of Science, Monash University, 18 Innovation Walk, Clayton, 3800 Melbourne, Australia; Hypertension Research Laboratory, School of Biological Sciences, Faculty of Science, Monash University, 18 Innovation Walk, Clayton, 3800 Melbourne, Australia; Hypertension Research Laboratory, School of Biological Sciences, Faculty of Science, Monash University, 18 Innovation Walk, Clayton, 3800 Melbourne, Australia; Hypertension Research Laboratory, School of Biological Sciences, Faculty of Science, Monash University, 18 Innovation Walk, Clayton, 3800 Melbourne, Australia; Heart Failure Research Group, Baker Heart and Diabetes Institute, 75 Commercial Road, 3004 Melbourne, Australia; Centre for Cardiovascular Biology and Disease Research (CCBDR), La Trobe Institute of Medical Science (LIMS), Bundoora, Victoria, Australia; Department of Microbiology, Anatomy, Physiology and Pharmacology, School of Agriculture, Biomedicine and Environment, La Trobe University, Bundoora, Victoria, Australia; Centre for Cardiovascular Biology and Disease Research (CCBDR), La Trobe Institute of Medical Science (LIMS), Bundoora, Victoria, Australia; Department of Microbiology, Anatomy, Physiology and Pharmacology, School of Agriculture, Biomedicine and Environment, La Trobe University, Bundoora, Victoria, Australia; Centre for Cardiovascular Biology and Disease Research (CCBDR), La Trobe Institute of Medical Science (LIMS), Bundoora, Victoria, Australia; Department of Microbiology, Anatomy, Physiology and Pharmacology, School of Agriculture, Biomedicine and Environment, La Trobe University, Bundoora, Victoria, Australia; Heart Failure Research Group, Baker Heart and Diabetes Institute, 75 Commercial Road, 3004 Melbourne, Australia; Department of Cardiology, Alfred Hospital, Melbourne, Australia; Central Clinical School, Faculty of Medicine Nursing and Health Sciences, Monash University, Melbourne, Australia; Infection and Immunity Program, Monash Biodiscovery Institute, Monash University, Melbourne, Australia; Department of Biochemistry, Monash University, Melbourne, Australia; School of Pharmaceutical Sciences, Shandong Analysis and Test Center, Qilu University of Technology (Shandong Academy of Sciences), Jinan 250014, China; Hypertension Research Laboratory, School of Biological Sciences, Faculty of Science, Monash University, 18 Innovation Walk, Clayton, 3800 Melbourne, Australia; Victorian Heart Institute, Monash University, 631 Blackburn Road, Clayton, 3800 Melbourne, Australia; Heart Failure Research Group, Baker Heart and Diabetes Institute, 75 Commercial Road, 3004 Melbourne, Australia

**Keywords:** Hypertension, Animal models, Microbiome, Microbiota

## Abstract

**Aims:**

Animal models are regularly used to test the role of the gut microbiome in hypertension. Small-scale pre-clinical studies have investigated changes to the gut microbiome in the angiotensin II hypertensive model. However, the gut microbiome is influenced by internal and external experimental factors, which are not regularly considered in the study design. Once these factors are accounted for, it is unclear if microbiome signatures are reproduceable. We aimed to determine the influence of angiotensin II treatment on the gut microbiome using a large and diverse cohort of mice and to quantify the magnitude by which other factors contribute to microbiome variations.

**Methods and results:**

We conducted a retrospective study to establish a diverse mouse cohort resembling large human studies. We sequenced the V4 region of the 16S rRNA gene from 538 samples across the gastrointestinal tract of 303 male and female C57BL/6J mice randomized into sham or angiotensin II treatment from different genotypes, diets, animal facilities, and age groups. Analysing over 17 million sequencing reads, we observed that angiotensin II treatment influenced α-diversity (*P* = 0.0137) and β-diversity (i.e. composition of the microbiome, *P* < 0.001). Bacterial abundance analysis revealed patterns consistent with a reduction in short-chain fatty acid producers, microbial metabolites that lower blood pressure. Furthermore, animal facility, genotype, diet, age, sex, intestinal sampling site, and sequencing batch had significant effects on both α- and β-diversity (all *P* < 0.001). Sampling site (6.8%) and diet (6%) had the largest impact on the microbiome, while angiotensin II and sex had the smallest effect (each 0.4%).

**Conclusion:**

Our large-scale data confirmed findings from small-scale studies that angiotensin II impacted the gut microbiome. However, this effect was modest relative to most of the other factors studied. Accounting for these factors in future pre-clinical hypertensive studies will increase the likelihood that microbiome findings are replicable and translatable.

## Graphical Abstract

**Graphical Abstract:**
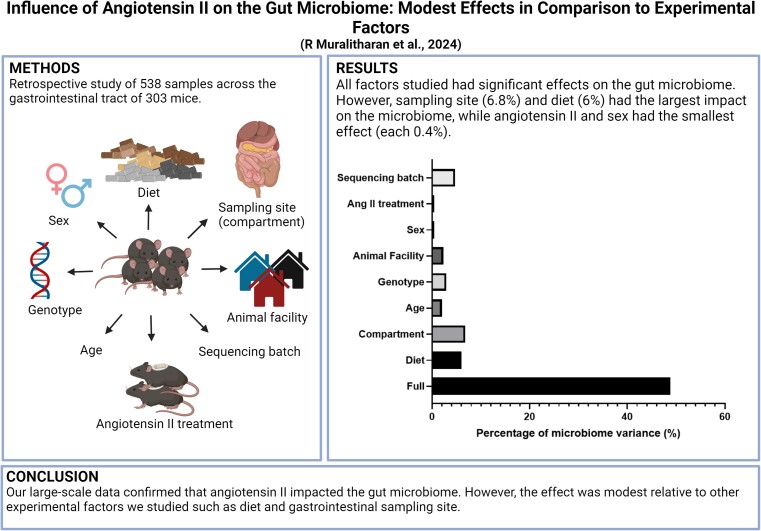

**Time of primary review: 36 days**

**See the editorial comment for this article ‘The significant impact of experimental variables on the gut microbiome', by B.I. Zakarauskas-Seth and S. Sawamiphak, https://doi.org/10.1093/cvr/cvae119.**

## 1. Introduction

In the last decade, the gut microbiome has emerged as a novel factor contributing to many health and pathological states, including hypertension. Small-scale case–control microbiome studies are regularly conducted in animal models—these are usually used as a proof of concept to show association between the gut microbiome and the disease of interest. However, regularly, these studies fail to account for internal, external, and technical factors that often co-influence the gut microbiome such as diet, living environment including breeding facilities, and cohort effects (see Supplementary material online, *Table S1*).^1–11^ These factors, if not accounted or adjusted for, could result in non-biologically relevant findings that are not replicable nor translatable. We know these factors influence the gut microbiome to a certain extent from previous smaller studies,^3^ but the combined magnitude of their impact is unclear. Similarly, human gut microbiome studies are often affected by many factors, which are nearly impossible to control at an experimental level. These external factors are often adjusted using normalization methods during data analysis.^12^ However, small-scale animal studies are usually under-powered to perform similar analyses.

The angiotensin II model is the most commonly used pre-clinical hypertensive model.^13^ In small-scale studies, angiotensin II treatment has previously been shown to influence the gut microbiome in mice.^14–16^ Here, we aimed to replicate the design of human populational studies in mice by performing a large retrospective study to determine the impact of angiotensin II on the gut microbiome. In our retrospective study, we included mouse samples from different diets and animal house facilities, ages, sex, cohorts, genotypes, and varied doses of angiotensin II (copying varied blood pressure levels as in humans). Using this heterogenous study cohort, we were able to determine what are the most important factors that influence the microbiome in an experimental hypertensive setting and provide robust evidence for an association between angiotensin II and specific microbial taxa.

## 2. Methods

### 2.1 Animals

Male and female mice [wild-type C57BL/6J (genotype 1) and knockout genotypes developed on the C57BL/6J background GPR41/43/109aKO (genotype 2), GPR41/43KO (genotype 3), and GPR65KO (genotype 4)], from three animal house facilities in Melbourne, Australia (animal facility 1, animal facility 2, and animal facility 3), were included in this study (*n* = 302; 253 male and 49 female mice). This is a retrospective study, and samples used came from independent studies where animal ethics approval was previously received. Mice were administered different diets [control 1 (AIN93G, Speciality Feeds); control 2 (chow, Barastoc); high-fibre (SF11-025, Speciality Feeds); no-fibre (SF09-028, Speciality Feeds); and high-amylose corn starch acetylated and butyrylated (30% HAMSAB, Ingredion, added to AIN93G)]. The only difference between the control 1, high-fibre, and no-fibre diets is the percentage of fibre, while protein, carbohydrate, and fat contents are similar across the diets. Some littermate mice were randomized and underwent minipump surgery containing angiotensin II (0.5 or 0.75 mg/kg body weight/day, *n* = 142) or 0.9% sodium chloride (sham, *n* = 45). This study also incorporated untreated mice (*n* = 116), which, along with the sham-operated mice, are collectively referred to as the sham group. Gut microbiota samples were collected from young (10–12 weeks, *n* = 264) and aged (6 months, *n* = 38) mice. Gut bacterial samples were collected from the caecum for all samples. Gastrointestinal contents for sequencing were also collected from the small intestine (separated into duodenum, jejunum, and ileum) and large intestine (caecum, colon, rectum, and faeces) from some mice (*n* = 13). The gastrointestinal contents were snap frozen in liquid nitrogen and stored at −80°C until further processing.

### 2.2 DNA extraction, library preparation, and sequencing

All samples were processed in the same laboratory. DNA was extracted using the DNeasy PowerSoil Kit (Qiagen) according to the manufacturer’s protocol. The V4 region of the bacterial 16S rRNA was amplified by PCR (Veriti Thermal Cycler, Thermo Fisher Scientific) using 20 ng of DNA, Platinum Hot Start PCR master mix (Thermo Fisher Scientific), 515F and 806R primers (Bioneer), single indexing, and methods as described in the Earth Microbiome Project.^17^ Thus, the microbiome data reported in this study refer to amplicon sequencing data. The quantity and quality of the PCR product were determined using a Qubit (Thermo Fisher Scientific). To identify contamination, non-template controls were used, and none showed amplification. PCR products of 240 ng per sample were pooled and cleaned using the PureLink PCR Purification kit (Thermo Fisher Scientific). Sequencing was performed across six batches (March 2020, October 2020, May 2021, August 2021, December 2021, and November 2022) in an Illumina MiSeq sequencer, generating 300 bp paired-end reads with 20% PhiX spiked in.

### 2.3 Bioinformatic and statistical analyses of gut microbiome

We used the QIIME2 framework to analyse the sequence reads.^18^ The forward and reverse reads were truncated at base number 220 for forward and 200 for reverse reads. The reads were then denoised, merged, and filtered for chimaera using the DADA2 plugin, resulting in an amplicon sequence variant (ASV) table at the single-nucleotide level.^19^ ASVs were then labelled by taxonomic assignment using a naïve Bayes classifier (via q2-feature-classifier),^20^ using the SILVA database (version 138) as a reference.^21^ The resulting taxonomic table was then uploaded to MicrobiomeAnalyst for downstream analyses.^22,23^ The data table was first filtered for low-prevalence (<20%) and low-variance (10% of interquartile range) reads. Data were then normalized using trimmed mean of *M*-values (TMM). The normalized data table was then used to generate two important indices commonly described in gut microbiome studies, the α- (a within-sample index) and β-diversity (a between-sample index). The α-diversity measure was determined using Shannon index, which is a more comprehensive metrics that determines both evenness and richness. α-Diversity is better explained by taking into consideration both evenness and richness compared to metrics that only consider evenness or richness; thus, Shannon index was chosen. Shannon index data were regraphed in GraphPad Prism version 9 and outliers removed using robust regression and outlier removal (1%). Two-tailed Mann–Whitney *U* test (for two groups) or Kruskal–Wallis (for three or more groups) with adjustment for false discovery rate (FDR) was performed. SPSS for Windows (release 25) was used for sensitivity analyses to determine which factors impacted Shannon index. A step-wise multiple linear regression model was used with all the factors as independent parameters (criteria of F-entry probability: 0.05, removal: 0.10). β-Diversity was determined using Bray–Curtis index, which takes into account the composition and abundance of the taxa. Differences between groups were assessed using permutational multivariate analysis of variance (PERMANOVA) for the overall effect. *P*-values of 0.05 were considered significant.

MaAsLin2 (in-built into MicrobiomeAnalyst) was used to determine multivariable association between taxa counts and the various experimental factors by generalized linear regression model using TMM normalized data.^24^ For the discovery of differentially abundant taxa, adjustment was made for animal house facility, genotype, diet, age, sex, sample acquisition site, treatment with angiotensin II (hypertension phenotype), and sequencing batch. FDR-adjusted *P*-values of <0.05 were considered significant. We performed a literature search of publicly available human gut microbiome database to cross-validate the differentially abundant taxa (species level) identified from MaAsLin2 with systolic blood pressure and plasma or faecal short-chain fatty acid (SCFA) levels. We performed simple nonparametric Spearman correlation of the identified taxa and systolic blood pressure. *P*-values of <0.05 were considered significant.

To determine the explained variance of each experimental factor on the gut microbiota composition in our study, we used VpThemAll.^25^ This package enabled us to determine the full, shared, and unique variance contributed by the eight experimental factors in our study. FDR-adjusted *P*-values of <0.05 were considered significant. Adjusted *r*^2^ values that were obtained were converted into percentage of variance.

### 2.4 Ethics approval

All samples used in this study were collected for independent studies where animal ethics approval was previously received. We retrospectively reanalysed all the samples.

## 3. Results

### 3.1 Sequencing reads and depth

**Figure 1:**
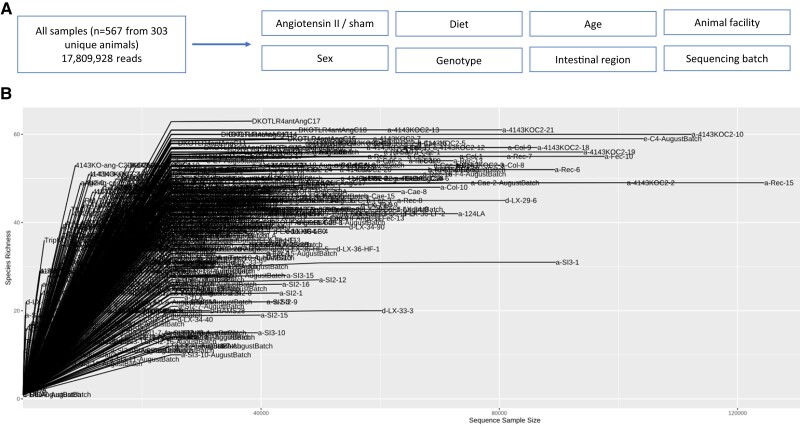
Study design and number of sequences per sample. (*A*) Brief schematic of study design. A total of 567 samples were sequenced from 303 animals, resulting in 17 809 928 reads. All samples were interrogated for various experimental factors: treatment of angiotensin II, diet, age, sex, genotype, intestinal region of sample collection site, animal facility, and sequencing batch. (*B*) Number of sequences per sample.

Over 17 809 928 reads were denoised, merged, and survived chimaera filtering, with read counts averaging 31 410 reads per sample (*Figure 1A* and *B*).

### 3.2 The impact of angiotensin II

**Figure 2:**
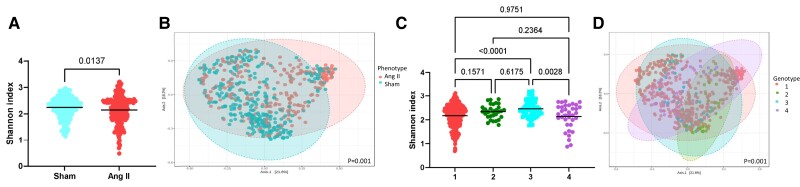
α- and β-diversity metrics of all samples (*n* = 538), categorized according to surgery (sham vs. angiotensin II; *A* and *B*) and genotypes 1–4, with 1 being wild type and 2–4 being knockout strains (*C* and *D*). (*A* and *C*) Shannon index, an α-diversity metric. (*B* and *D*) Bray–Curtis index (β-diversity metric). Significance of Shannon index (*P* < 0.05) determined using Mann–Whitney *U* (two comparison groups) or Kruskal–Wallis (more than two comparison groups) tests. Significance of Bray–Curtis index determined using PERMANOVA with adjustment for multiple comparisons (*q* < 0.05).

We investigated whether high blood pressure induced by angiotensin II resulted in differences in the gut microbiota. We found significant differences in both α- (*P* = 0.0137, *Figure 2A*) and β-diversity (*P* = 0.001, *Figure 2B*). We identified 20 significantly differentially abundant taxa including *Clostridium leptum* (see Supplementary material online, *Table S2*)—known to produce SCFAs, gut microbiota–derived metabolites shown to lower blood pressure in mice and hypertensive patients.^16,26–28^ In a publicly available human hypertension cohort database in which *C. leptum* was identified,^29^*C. leptum* abundance negatively correlated to systolic blood pressure (*P* = 0.0145; Supplementary material online, *Figure S1*). In a recent publication in which *C. leptum* abundance positively correlated with plasma butyric acid levels in a cohort 241 participants, confirming its function as a SCFA (butyrate) producer.^30^ In this study however, shotgun metagenomics was performed, which enhanced the identification of *C. leptum*.

### 3.3 The impact of the genotype

We next examined whether the mouse genotype influenced the gut bacterial metrics. The various mouse genotypes used in this study were aimed to introduce and increase genetic variation in the host to assess their role on the gut microbiome. This is important because essential hypertension is a multifactorial condition.^31^ We observed some significant changes in α-diversity, particularly between genotypes 1 and 3 (*P* < 0.0001) and genotypes 3 and 4 (*P* = 0.0028, *Figure 2C*). There was also a significant change in β-diversity (*P* = 0.001, *Figure 2D*), with some genotypes (e.g. genotype 2) being the most dissimilar across groups. Pairwise comparisons for β-diversity metrics reveal all four genotypes are significantly different to each other (see Supplementary material online, *Table S3*). We identified 116 taxa that were different across the genotypes (see Supplementary material online, *Table S4*), particularly in mice lacking GPR41/43/109A, which lack the three main receptors that sense SCFAs.

### 3.4 The impact of animal house facilities

**Figure 3:**
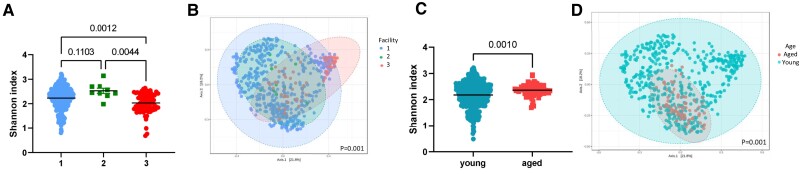
α- and β-diversity metrics of all samples (*n* = 538), categorized according to animal facility (*A* and *B*) and age group (*C* and *D*). (*A* and *C*), Shannon index, an α-diversity metric. (*B* and *D*) Bray–Curtis index (β-diversity metric). Significance of Shannon index (*P* < 0.05) determined using Mann–Whitney *U* (two comparison groups) or Kruskal–Wallis (more than two comparison groups) tests. Significance of Bray–Curtis index determined using PERMANOVA with adjustment for multiple comparisons (*q* < 0.05).

We also studied the influence of animal house facilities by using samples from three distinct facilities in Australia. We determined that animal house facility influences bacterial diversity, with differences in both Shannon index (*P* = 0.0012–0.1103, *Figure 3A*) and Bray–Curtis index (*P* = 0.001, *Figure 3B*)—these show distinct clustering according to the origin of the mice. Pairwise comparisons revealed significant differences between all three facilities in Bray–Curtis index (see Supplementary material online, *Table S5*). We identified 95 bacteria taxa that were significantly different between animal houses (see Supplementary material online, *Table S6*). This included common gut bacteria, such as those from the genera *Bacteroides*, *Alistipes*, *Mucispirillum*, and *Azospirillum*.

### 3.5 The impact of age

We categorized the samples into young (10–12 weeks) and aged (6 months) groups based on age. Although there was a large difference in sample size between young and aged mice, we found that aged mice had higher α-diversity (*P* < 0.0001, *Figure 3C*) and distinct β-diversity metrics compared to younger mice (*P* = 0.001, *Figure 3D*). We also identified 53 taxonomic changes between young and aged mice, with younger mice having higher prevalence of SCFA producers such as *Ruminococcus* and *Blautia* compared to aged mice (see Supplementary material online, *Table S7*).

### 3.6 The impact of sex

**Figure 4:**
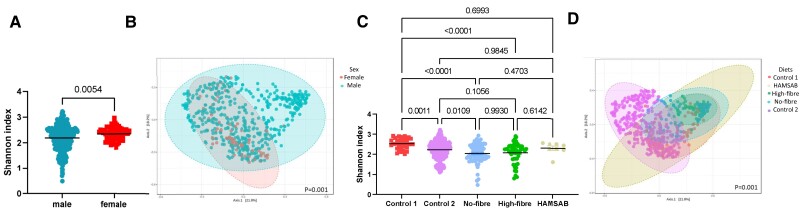
α- and β-diversity metrics of all samples (*n* = 538), categorized according to sex (*A* and *B*) and diets (*C* and *D*). (*A* and *C*) Shannon index, an α-diversity metric. (*B* and *D*) Bray–Curtis index (β-diversity metric). Significance of Shannon index (*P* < 0.05) determined using Mann–Whitney *U* (two comparison groups) or Kruskal–Wallis (more than two comparison groups) tests. Significance of Bray–Curtis index determined using PERMANOVA with adjustment for multiple comparisons (*q* < 0.05).

There is growing interest in the representation of both male and female mice in animal studies; however, it is still unclear if there are differences in the gut microbiota driven by sex.^32^ We found that male and female mice did not differ significantly in α-diversity (*P* = 0.054, *Figure 4A*), but differed in terms of β-diversity metrics (*Figure 4B*). Albeit not distinct in the principal coordinates analysis (PCoA) plots, sex reached statistical significance (*P* = 0.001), with 35 taxonomic changes between the sexes (see Supplementary material online, *Table S8*). Male mice had lower levels of SCFA-producing bacteria including *Roseburia*, *Blautia*, and *Lachnospiraceae bacterium*, relative to female mice.

### 3.7 The impact of diet

Diet is a well-known factor that impacts the microbiome.^33^ We investigated the influence of diet using five different diets, four of which were manipulated in the same background, so the only difference was the amount and type of dietary fibre, a key prebiotic nutrient that feeds the microbiota. These diets had a clear impact on the microbiome—we found some differences in α-diversity particularly between control 1 and high-fibre diet (*P* < 0.0001) and control 1 and low-fibre diet (*P* < 0.0001, *Figure 4C*), with an overall difference in β-diversity (*P* = 0.001, *Figure 4D*). Pairwise comparisons for β-diversity metrics reveal all diets are mostly different from each other (see Supplementary material online, *Table S9*). Importantly, there was a significant difference in both α- and β-diversity between the two control diets, showing the importance for control diets to be nutrient matched. The different diets modulated 192 taxa (see Supplementary material online, *Table S10*), with a no-fibre diet being the major driver of these changes (74 out of the 192 taxa). The no-fibre diet reduced the prevalence of bacteria known to ferment fibre and produce SCFAs, such as *Roseburia* and *Lachnospiraceae NK4A136 group*, while a high-fibre diet increased the prevalence of genus *Akkermansia*.

### 3.8 The impact of gastrointestinal tissue sampling

**Figure 5:**
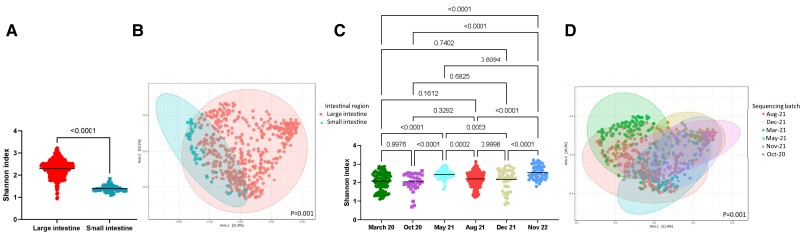
α- and β-diversity metrics of all samples (*n* = 538), categorized according to intestinal region (*A* and *B*) and sequencing batch (*C* and *D*). (*A* and *C*) Shannon index, an α-diversity metric. (*B* and *D*) Bray–Curtis index (β-diversity metric). Significance of Shannon index (*P* < 0.05) determined using Mann–Whitney *U* (two comparison groups) or Kruskal–Wallis (more than two comparison groups) tests. Significance of Bray–Curtis index determined using PERMANOVA with adjustment for multiple comparisons (*q* < 0.05).

Due to differences in pH and bacterial density,^34^ samples from the large and small intestines were expected to differ. We confirmed that the small intestine had lower α-diversity (*P* < 0.0001, *Figure 5A*) and different β-diversity metrics compared to the large intestine (*P* = 0.001, *Figure 5B*). We also observed 72 taxa that changed between these intestinal regions (see Supplementary material online, *Table S11*), with the small intestine having lower prevalence of 63 out of the 72 taxa.

### 3.9 The impact of sequencing batch effect

Sequencing batch effect is a well-known issue in which technical variations involved with sample preparation and sequencing platform results in differences in terms of bacteria identified.^35^ This poses a problem when sequenced data are combined, often observed in large-scale human studies.^36^ Samples in our study were sequenced across six batches. We found that both α- (*P* = 0.0001–0.9998, *Figure 5C*) and β-diversity metrics (*P* = 0.001, *Figure 5D*; Supplementary material online, *Table S12*) were significantly influenced by sequencing batch effect. This also resulted in 243 differential taxa across the batches (see Supplementary material online, *Table S13*).

### 3.10 Degree of variation contributed by experimental factors

**Figure 6.**
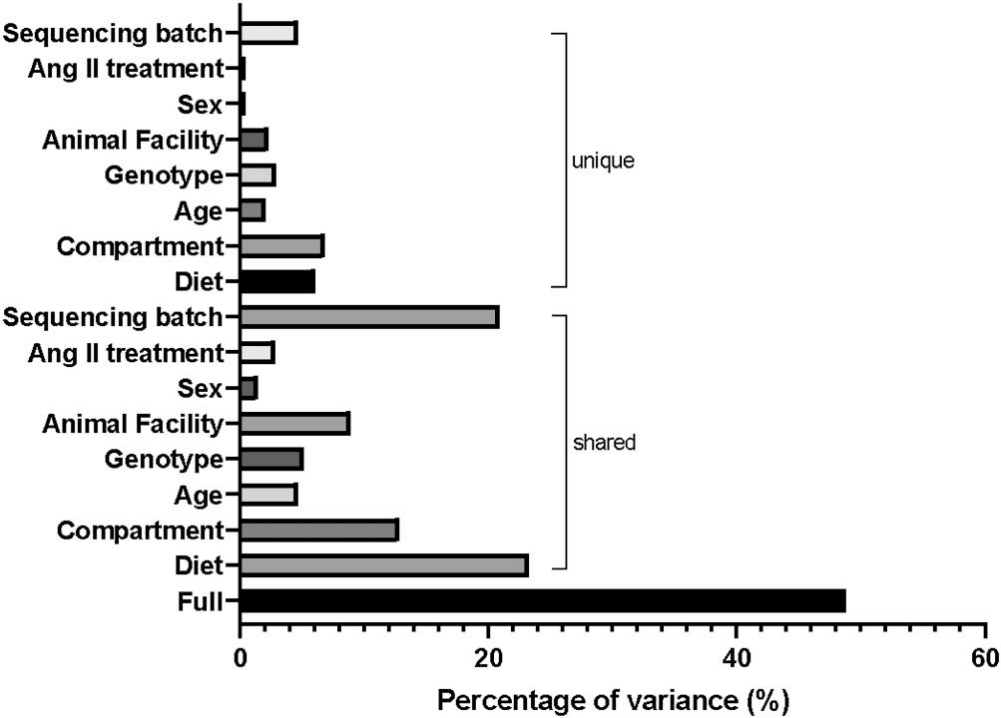
The full, shared, and unique contribution of each experimental factor to the variations in the gut microbiota composition. Full represents how much all the experimental factors in our study explain the variations observed in the gut microbiome. Shared represents how much each factor explains the variation without correcting for the other variables. Unique is how much each factor explains the variations observed, after correcting for all other variables (refer to Supplementary material online, *Table S14*, for further information).

We leverage a tool called VPThemAll^25^ to determine the contribution of these various factors to the variability observed in the gut microbiome. We identified that all the factors investigated in this study significantly contributed to gut microbial variations (*Figure 6*; Supplementary material online, *Table S14*). The full effects of all the factors combined explained 48.8% of variations in the gut microbiome (see Supplementary material online, *Table S14*). Shared variances explain how much variations observed in the gut microbiome are due to each experimental factor, without correction for the other variables (see Supplementary material online, *Table S14*). Diet had the largest influence in the shared element of the data (23.3%). The unique element, which is the variation contributed by a single factor following adjustment for all the factors, was largely contributed by the compartment from which the sample was acquired from (6.8%), followed by diet (6%), and then sequencing batch (4.7%; see Supplementary material online, *Table S14*). Age, genotype, and animal facility each explained ∼2% of variance in the gut microbial composition (see Supplementary material online, *Table S14*). Sex and the treatment of angiotensin II had minimal effects on the gut microbial composition, each explaining 0.4% of the variance (see Supplementary material online, *Table S14*).

We also performed a step-wise multiple regression analysis to determine which were the key factors drive α-diversity. Gastrointestinal compartment (*β* = −0.822, 95% CI: −0.939 to −0.706, *P* < 0.001), animal house facility (*β* = −0.227, 95% CI: −0.309 to −0.145, *P* < 0.001), and age group (*β* = −0.132, 95% CI: 0.032–0.232, *P* = 0.009) were the only factors associated with Shannon index in our cohort. Gastrointestinal compartment had the largest impact (standardized *β* = −0.503) relative to facility (standardized *β* = −0.197) and age group (standardized *β* = 0.094).

## 4. Discussion

We performed a retrospective study to determine the role of angiotensin II and several other experimental factors on the gut microbiome of laboratory mice. We sequenced gut microbiome samples from a heterogenous cohort of mice to introduce the influence of external factors such as diet, living environment, differences in blood pressure, genetics/genotype, sex, and age. Despite the presence of confounding factors, our study findings are in agreement with previous small-scale studies showing that angiotensin II influenced microbial diversity.^14–16,37^ Despite the influence of confounding factors, normalized data in a highly powered cohort found a few differentially abundant bacteria in common with previous studies. However, we determined that only 0.4% of the gut microbial composition differences were due to the treatment with angiotensin II, and angiotensin II was not associated with α-diversity when all factors were considered. Our findings also revealed that diet and intestinal region where samples were collected from had a large influence on the variations in the gut microbiome. Lack of appropriate control of those may lead to misleading findings that could be attributed to disease phenotypes such as angiotensin II.

The gut microbiome is often influenced by many external confounding factors, which are often difficult to be controlled for. To control for these factors at the experimental level, many animals would be required, which is not feasible or ethical. One of the approaches to overcome this issue is to validate findings across different facilities or by performing meta-analysis of multiple studies. We took advantage of the availability of samples processed by the same laboratory and team, but from across multiple facilities and cohorts with the influence of many external factors. To our knowledge, this is the largest sample size ever analysed in laboratory mouse gut microbiome (see Supplementary material online, *Table S1*). The findings of our study highlight the potential of using a retrospective approach of a heterogenous cohort of laboratory animals as a validation method of small-scale pre-clinical animal models given that factors influencing the gut microbiome are reported.

Our study also highlights that there are many confounding factors that influence the variations observed in the gut microbiome. In addition, we were able to determine the magnitude by which these factors influence the gut microbiome—unsurprisingly, diet and the intestinal region where the sample was collected were the largest contributors to the variations observed in the gut microbiome. The findings support guidelines for gut microbiome studies in hypertension and renal function, where appropriate control for these factors has been suggested.^38,39^

In our study, we investigated the bacterial component of the gut microbiome as a proof of concept. However, how signatures of other members of the gut microbiome such as phages and fungi are affected by external factors remains unclear. The shared contribution of the factors we investigated only accounted for about 50% of the observed variation in the gut microbiome, meaning other factors are also at play. Other experimental factors not studied here may also impact the gut microbiome of experimental animals. This includes animal vendor,^40^ which could not be addressed in our study as all animals included were bred in-house in each of the three facilities studied. Other factors, which were not considered in this study, include cage mate effect prior to surgery and bedding, and despite the fact that the mice are from specific pathogen-free facilities, the presence of different pathogens can influence the gut microbiome too.^6,41,42^ How these factors interact with each other is also unclear.

We found that <1% of the gut microbiome was influenced by the treatment of angiotensin II. This suggests that if experimental groups are not appropriately controlled by other factors known to influence the gut microbiome, a false-positive association is likely. An example is the significant difference between the two control diets we compared. In addition, any study with sufficient sample size would detect a statistical association, but whether this is of biological significance depends on the study design.

In future studies, it would be interesting to determine the magnitude that dietary salt contributes to variations in the gut microbiome in addition to angiotensin II, as well as a meta-analysis to compare the gut microbiome across different models of hypertension. In these future studies, it is important to investigate blood pressure as an outcome. At the moment, this is a limitation of the field as many studies did not make microbiome data publicly available.

In conclusion, our large-scale retrospective study validated findings from small-scale studies that angiotensin II treatment influences the gut microbiome; however, this effect was very small relative to most of the other factors studied. Our findings also demonstrate the potential use of a heterogenous animal cohort to retrospectively investigate the role of the gut microbiome in pre-clinical disease models while being able to control for confounding variables. Controlling for these factors in future pre-clinical studies in experimental hypertension will increase the likelihood that microbiome findings are replicable and translatable. This approach could be used for other cardiovascular disease models to validate gut microbiome findings from small-scale animal studies.

## Supplementary Material

cvae062_Supplementary_Data

## Data Availability

Sequencing data and metadata file are publicly available at https://doi.org/10.5281/zenodo.7935261.
