# Supplementary material for: Influence of angiotensin II on the gut microbiome: modest effects in comparison to experimental factors
Source: Cardiovasc Res. 2024 Mar 22;120(10):1155–63. doi: 10.1093/cvr/cvae062 (PMC11368123; doi:10.1093/cvr/cvae062)
Supplement: cvae062_Supplementary_Data [file cvae062_supplementary_data.docx]

**Online supplementary file to**

**Influence of angiotensin II on the gut microbiome: modest effects in comparison to experimental factors**

**Short title:** Gut Microbiome in Angiotensin II Model

Rikeish R. Muralitharan^1,2^, Michael E. Nakai^1^, Matthew Snelson^1,3^, Tenghao Zheng^1^, Evany Dinakis^1^, Liang Xie^1^, Hamdi Jama^1^, Madeleine Paterson^1^, Waled Shihata^4^, Flavia Wassef^5^, Antony Vinh^5^, Grant R Drummond^5^, David M. Kaye^4,6,7^, Charles R. Mackay^8,9,10^,

Francine Z. Marques^1,3,4^*

^1^Hypertension Research Laboratory, School of Biological Sciences, Faculty of Science, Monash University, Melbourne, Australia; ^2^Institute for Medical Research, Ministry of Health Malaysia, Kuala Lumpur, Malaysia; ^3^Victorian Heart Institute, Monash University, Melbourne, Australia.; ^4^Heart Failure Research Group, Baker Heart and Diabetes Institute, Melbourne, Australia; ^5^Department of Microbiology, Anatomy, Physiology, La Trobe University, Melbourne, Australia; ^6^Department of Cardiology, Alfred Hospital, Melbourne, Australia; ^7^Central Clinical School, Faculty of Medicine Nursing and Health Sciences, Monash University, Melbourne, Australia;^8^Infection and Immunity Program, Monash Biodiscovery Institute, Monash University, Melbourne, Australia; ^9^Department of Biochemistry, Monash University, Melbourne, Australia; ^10^School of Pharmaceutical Sciences, Shandong Analysis and Test Center, Qilu University of Technology (Shandong Academy of Sciences), Jinan, 250014, China;

***Correspondence to**: A/Prof Francine Marques. Hypertension Research Laboratory, School of Biological Sciences, Monash University, 25 Rainforest Walk, Clayton, Victoria 3800, Australia. P: +61-03-99056958. E: Francine.marques@monash.edu

**Supplementary Figure 1**

**Figure S1.** *Clostridium leptum* relative abundance (%) correlates to systolic blood pressure (mmHg) in a human cohort of 31 hypertensives and 10 control participants (P-0.0145). Correlation performed using non-parametric Spearman, P-values <0.05 were considered significant.

**Supplementary Tables**

**Table S1.** Summary of previous studies that investigated the role of experimental factors that influence the gut bacterial diversity metrics in laboratory mice. Search performed on PubMed on 9^th^ March 2023 using the following keywords: ‘alpha diversity’, ‘beta diversity’, ‘experimental factors’, ‘laboratory’, ‘mice’, ‘animals’.

| **Study** | **Factor(s) investigated** | **Total sample size** | **Findings** |
| --- | --- | --- | --- |
| Turnbaugh et al., 2008 | Diet | 10 | Conventionalised germ-free mice fed two different diets had significant difference in β-diversity |
| Hufeldt et al., 2010 | Mice vendor | 33 | C57BL/6 mice from 2 different vendors had different caecal microbiota composition |
|  | Different rooms in same breeding center | 33 | C57BL/6 mice from 1 vendor bred in two different rooms in the same breeding centre had similar microbiota composition |
|  | Sex | 33 | No difference between male and female mice from same vendor housed in the same room but different cages |
| Hildebrand et al., 2013 | Genetic effects | 50 | Genetic effects influenced up to 15.65 to 18.62% of variations at lower than phylum levels among 4 genotypes |
|  | Cage effects | 50 | Up to 30% of variations in the gut microbiome were explained by cage effects |
| Rausch et al., 2016 | Housing type | 94 | Difference in α- and β-diversity in mice housed in IVC compared to others |
|  | Chow treatment | 104 | Difference in α- and β-diversity in autoclaved food compared to irradiated and untreated |
|  | Chow provider | 104 | No difference in α-diversity between two chow providers, but different β-diversity |
| Hilbert et al., 2017 | Vendor effects | 60 | C57BL/6 from three vendors had distinct gut microbial composition |
| Ericsson et al., 2018 | Cage ventilation and bedding | 144 | Cage ventilation and bedding and the interaction, had a distinguishable effect on the caecal microbiota but not in the faecal or small intestine samples |
| Montonye et al., 2018 | Transport | 16 | Significant difference in α- and β-diversity pre- and post-arrival |
|  | Animal facility | 8 | The α- and β-diversity of mice pre-shipping is significantly different to microbiota upon arrival at facilities and between facilities |
| Robertson et al., 2019 | Mice vendor | 8 | Mice from 2 vendors had no difference in α-diversity but significantly different β-diversity. Samples from pellet, colon, ileum |
|  | Littermate effect and co-housing effect | 28 | Mice that were co-housed had similar gut microbiome to each other but also their non-co-housed siblings (n=12) in the colon and the ileum but not in the faecal pellet. |
| Wolff et al., 2020 | Vendor effects | 24 | Significant difference in β-diversity of genetically identical C57BL/6 from three different vendors |
| Singh et al., 2021 | Cage and maternal effects | 30 | The gut microbiome of F2 littermates of different genotypes (n=10 per group) from heterozygous parents (mdr1a-/+) clustered according to their maternal origin and cages. |
| Guo et al., 2022 | Mouse genotypes | 110 | Significant difference in α- and β-diversity of 7 background genotypes of C57BL/6 and BALB/c mice |

**Table S2**. Differences in gut microbiome taxa between angiotensin II treatment relative to sham, showing taxa with q<0.05.

| **Taxa** | **Log2FC** | **St.Error** | **P-value** | **FDR** |
| --- | --- | --- | --- | --- |
| Sham/Angiotensin II |  |  |  |  |
| p__Firmicutes | 0.422 | 0.145 | 0.00385 | 0.0101 |
| c__Gammaproteobacteria | 0.896 | 0.248 | 3.22E-04 | 0.00121 |
| o__Burkholderiales | 0.896 | 0.248 | 3.22E-04 | 0.0013 |
| o__Erysipelotrichales | 0.82 | 0.267 | 0.00225 | 0.00698 |
| o__Oscillospirales | 0.599 | 0.206 | 0.00377 | 0.0112 |
| f__Sutterellaceae | 0.896 | 0.248 | 3.22E-04 | 0.00131 |
| f__Erysipelotrichaceae | 0.82 | 0.267 | 0.00225 | 0.00719 |
| f__Oscillospiraceae | 0.596 | 0.207 | 0.00418 | 0.0126 |
| g__Oscillibacter | 0.985 | 0.241 | 5.15E-05 | 2.52E-04 |
| g__Parasutterella | 0.896 | 0.248 | 3.22E-04 | 0.00127 |
| g__uncultured | 0.537 | 0.19 | 0.00494 | 0.0141 |
| g__Colidextribacter | 0.653 | 0.237 | 0.00605 | 0.0167 |
| g__Lachnoclostridium | 0.641 | 0.235 | 0.00664 | 0.018 |
| g__ASF356 | 0.619 | 0.261 | 0.0179 | 0.042 |
| g__Bilophila | 0.536 | 0.228 | 0.019 | 0.0443 |
| Not_Assigned | 0.369 | 0.159 | 0.021 | 0.0487 |
| g__Desulfovibrio | 0.543 | 0.236 | 0.0216 | 0.0499 |
| unidentified | 0.68 | 0.224 | 0.00246 | 0.00716 |
| Clostridium_leptum | 0.693 | 0.244 | 0.00466 | 0.0128 |
| uncultured_bacterium | 0.192 | 0.084 | 0.0227 | 0.0468 |

**Legend:** d (domain), p (phylum), c (class), o (order), f (family), g (genus), standard error (St. Error), false-discovery rate adjusted p-value (FDR)

**Table S3**. Pairwise comparisons between the genotypes for Bray-Curtis index (a metric of β-diversity).

| **Comparisons** | **F-value** | **R-squared** | **P-value** | **FDR** |
| --- | --- | --- | --- | --- |
| Genotype 3 vs Genotype 1 | 5.266 | 0.010422 | 0.001 | 0.001 |
| Genotype 3 vs Genotype 4 | 9.2001 | 0.087454 | 0.001 | 0.001 |
| Genotype 3 vs Genotype 2 | 15.509 | 0.14561 | 0.001 | 0.001 |
| Genotype 1 vs Genotype 4 | 5.3561 | 0.01122 | 0.001 | 0.001 |
| Genotype 1 vs Genotype 2 | 15.844 | 0.032814 | 0.001 | 0.001 |
| Genotype 4 vs Genotype 2 | 13.851 | 0.18023 | 0.001 | 0.001 |

**Table S4**. Differences in gut microbiome taxa across genotypes relative to wild-type mice (Genotype #1) (showing taxa with q<0.05).

| Name | Log2FC | St.Error | P-value | FDR |
| --- | --- | --- | --- | --- |
| WT/GPR41-43-109aKO |  |  |  |  |
| p__Bacteroidota | 0.664 | 0.125 | 1.48E-07 | 1.17E-06 |
| p__Verrucomicrobiota | -3.27 | 0.714 | 5.75E-06 | 3.15E-05 |
| p__Deferribacterota | 2.47 | 0.694 | 4.15E-04 | 0.00141 |
| p__Firmicutes | -0.966 | 0.294 | 0.00108 | 0.00324 |
| c__Bacteroidia | 0.664 | 0.125 | 1.48E-07 | 1.41E-06 |
| c__Bacilli | -2.93 | 0.619 | 2.74E-06 | 1.77E-05 |
| c__Verrucomicrobiae | -3.27 | 0.714 | 5.75E-06 | 3.32E-05 |
| c__Deferribacteres | 2.47 | 0.694 | 4.15E-04 | 0.00146 |
| o__Bacteroidales | 0.664 | 0.125 | 1.48E-07 | 1.25E-06 |
| o__Verrucomicrobiales | -3.27 | 0.714 | 5.75E-06 | 3.61E-05 |
| o__Acholeplasmatales | -3.7 | 0.872 | 2.59E-05 | 1.40E-04 |
| o__Clostridia_vadinBB60_group | -1.81 | 0.496 | 2.87E-04 | 0.00117 |
| o__Deferribacterales | 2.47 | 0.694 | 4.15E-04 | 0.00165 |
| o__Erysipelotrichales | -1.75 | 0.54 | 0.00123 | 0.00405 |
| o__Lachnospirales | -0.986 | 0.399 | 0.0137 | 0.0353 |
| f__Rikenellaceae | 4.07 | 0.522 | 3.24E-14 | 7.14E-13 |
| f__Eubacterium_coprostanoligenes_group | 3.23 | 0.433 | 3.58E-13 | 6.44E-12 |
| f__Bacteroidaceae | 1.5 | 0.302 | 9.42E-07 | 7.18E-06 |
| f__Akkermansiaceae | -3.27 | 0.714 | 5.75E-06 | 3.67E-05 |
| f__Acholeplasmataceae | -3.7 | 0.872 | 2.59E-05 | 1.43E-04 |
| f__Clostridia_vadinBB60_group | -1.81 | 0.496 | 2.87E-04 | 0.00118 |
| f__Deferribacteraceae | 2.47 | 0.694 | 4.15E-04 | 0.00164 |
| f__Erysipelotrichaceae | -1.75 | 0.54 | 0.00123 | 0.00413 |
| f__Lachnospiraceae | -0.986 | 0.399 | 0.0137 | 0.0351 |
| g__Muribaculum | -5.48 | 0.516 | 4.44E-24 | 1.68E-22 |
| g__Dubosiella | -3.06 | 0.365 | 4.45E-16 | 1.14E-14 |
| g__Alistipes | 4.07 | 0.522 | 3.24E-14 | 7.30E-13 |
| g__Eubacterium_coprostanoligenes_group | 3.23 | 0.433 | 3.58E-13 | 7.36E-12 |
| g__Ileibacterium | -2.92 | 0.557 | 2.34E-07 | 1.89E-06 |
| g__Eubacterium_xylanophilum_group | 2.25 | 0.451 | 8.14E-07 | 6.01E-06 |
| g__Bacteroides | 1.5 | 0.302 | 9.42E-07 | 6.85E-06 |
| g__GCA_900066575 | -2.54 | 0.514 | 1.01E-06 | 7.27E-06 |
| g__Akkermansia | -3.27 | 0.714 | 5.75E-06 | 3.51E-05 |
| g__Anaerotruncus | 2.56 | 0.566 | 7.48E-06 | 4.49E-05 |
| g__Tuzzerella | -1.99 | 0.466 | 2.23E-05 | 1.15E-04 |
| g__Anaeroplasma | -3.7 | 0.872 | 2.59E-05 | 1.31E-04 |
| g__Intestinimonas | 2.27 | 0.546 | 3.71E-05 | 1.84E-04 |
| g__Desulfovibrio | 1.82 | 0.477 | 1.46E-04 | 6.03E-04 |
| g__Bilophila | -1.69 | 0.461 | 2.59E-04 | 0.00104 |
| g__Clostridia_vadinBB60_group | -1.81 | 0.496 | 2.87E-04 | 0.00112 |
| g__Mucispirillum | 2.47 | 0.694 | 4.15E-04 | 0.00155 |
| g__Lachnospiraceae_UCG_006 | -1.81 | 0.512 | 4.28E-04 | 0.00159 |
| g__Dorea | 1.55 | 0.442 | 4.74E-04 | 0.00172 |
| g__Lachnospiraceae_NK4A136_group | -1.36 | 0.484 | 0.00509 | 0.014 |
| uncultured_Barnesiella | 6.87 | 0.481 | 1.39E-39 | 9.17E-38 |
| Dubosiella_newyorkensis | -3.06 | 0.365 | 4.45E-16 | 7.34E-15 |
| uncultured_Bacteroidales | 2.09 | 0.297 | 6.14E-12 | 6.75E-11 |
| Clostridium_leptum | -2.66 | 0.493 | 9.58E-08 | 6.33E-07 |
| Ileibacterium_valens | -2.92 | 0.557 | 2.34E-07 | 1.40E-06 |
| mouse_gut | 2.25 | 0.483 | 3.87E-06 | 1.92E-05 |
| unidentified | -1.27 | 0.452 | 0.00525 | 0.0128 |
| WT/GPR41-43KO |  |  |  |  |
| p__Deferribacterota | 4.21 | 0.49 | 9.32E-17 | 2.35E-15 |
| p__Verrucomicrobiota | -2.49 | 0.504 | 1.07E-06 | 7.52E-06 |
| p__Proteobacteria | -1.21 | 0.357 | 7.17E-04 | 0.00232 |
| c__Deferribacteres | 4.21 | 0.49 | 9.32E-17 | 3.02E-15 |
| c__Verrucomicrobiae | -2.49 | 0.504 | 1.07E-06 | 7.91E-06 |
| c__Alphaproteobacteria | -1.6 | 0.483 | 0.00101 | 0.00326 |
| o__Deferribacterales | 4.21 | 0.49 | 9.32E-17 | 2.80E-15 |
| o__Verrucomicrobiales | -2.49 | 0.504 | 1.07E-06 | 8.05E-06 |
| o__Clostridia_vadinBB60_group | -1.26 | 0.351 | 3.71E-04 | 0.00152 |
| o__Rhodospirillales | -1.6 | 0.483 | 0.00101 | 0.00357 |
| o__Peptococcales | 0.762 | 0.288 | 0.00846 | 0.0246 |
| f__Eubacterium_coprostanoligenes_group | 3.55 | 0.306 | 5.24E-28 | 1.73E-26 |
| f__Deferribacteraceae | 4.21 | 0.49 | 9.32E-17 | 2.17E-15 |
| f__Akkermansiaceae | -2.49 | 0.504 | 1.07E-06 | 8.18E-06 |
| f__Clostridia_vadinBB60_group | -1.26 | 0.351 | 3.71E-04 | 0.00155 |
| f__Tannerellaceae | -0.98 | 0.292 | 8.32E-04 | 0.00314 |
| f__uncultured | -1.6 | 0.483 | 0.00101 | 0.00366 |
| f__Peptococcaceae | 0.762 | 0.288 | 0.00846 | 0.0245 |
| g__Eubacterium_coprostanoligenes_group | 3.55 | 0.306 | 5.24E-28 | 2.36E-26 |
| g__Mucispirillum | 4.21 | 0.49 | 9.32E-17 | 2.49E-15 |
| g__Dubosiella | -1.95 | 0.258 | 1.60E-13 | 3.39E-12 |
| g__Akkermansia | -2.49 | 0.504 | 1.07E-06 | 8.40E-06 |
| g__Faecalibaculum | 1.35 | 0.368 | 2.67E-04 | 0.00117 |
| g__Clostridia_vadinBB60_group | -1.26 | 0.351 | 3.71E-04 | 0.00153 |
| g__Muribaculum | -1.24 | 0.364 | 7.08E-04 | 0.00273 |
| g__A2 | 1.33 | 0.415 | 0.00136 | 0.00462 |
| g__Bilophila | -0.912 | 0.325 | 0.00523 | 0.0154 |
| g__Eubacterium_xylanophilum_group | -0.85 | 0.318 | 0.00777 | 0.0216 |
| g__Tuzzerella | -0.806 | 0.329 | 0.0146 | 0.0372 |
| Dubosiella_newyorkensis | -1.95 | 0.258 | 1.60E-13 | 2.64E-12 |
| unidentified | -1.3 | 0.319 | 5.54E-05 | 2.39E-04 |
| uncultured_bacterium | 0.357 | 0.12 | 0.00305 | 0.00863 |
| Not_Assigned | -0.226 | 0.0886 | 0.0109 | 0.0247 |
| Lachnospiraceae_bacterium | -0.942 | 0.416 | 0.0241 | 0.0478 |
| WT/GPR65KO |  |  |  |  |
| p__Verrucomicrobiota | -3.77 | 0.659 | 1.66E-08 | 1.61E-07 |
| c__Verrucomicrobiae | -3.77 | 0.659 | 1.66E-08 | 1.80E-07 |
| c__Alphaproteobacteria | 1.98 | 0.632 | 0.00184 | 0.00575 |
| o__Verrucomicrobiales | -3.77 | 0.659 | 1.66E-08 | 1.66E-07 |
| o__Clostridia_vadinBB60_group | 2.37 | 0.458 | 3.12E-07 | 2.41E-06 |
| o__Clostridia_UCG_014 | -2 | 0.527 | 1.66E-04 | 7.01E-04 |
| o__Rhodospirillales | 1.98 | 0.632 | 0.00184 | 0.00607 |
| f__Akkermansiaceae | -3.77 | 0.659 | 1.66E-08 | 1.61E-07 |
| f__Eubacterium_coprostanoligenes_group | -2.1 | 0.4 | 2.22E-07 | 1.79E-06 |
| f__Clostridia_vadinBB60_group | 2.37 | 0.458 | 3.12E-07 | 2.42E-06 |
| f__Clostridia_UCG_014 | -2 | 0.527 | 1.66E-04 | 7.15E-04 |
| f__uncultured | 1.98 | 0.632 | 0.00184 | 0.00614 |
| g__Akkermansia | -3.77 | 0.659 | 1.66E-08 | 1.87E-07 |
| g__Eubacterium_coprostanoligenes_group | -2.1 | 0.4 | 2.22E-07 | 1.88E-06 |
| g__Clostridia_vadinBB60_group | 2.37 | 0.458 | 3.12E-07 | 2.58E-06 |
| g__GCA_900066575 | -2.28 | 0.475 | 1.96E-06 | 1.41E-05 |
| g__Faecalibaculum | 2.2 | 0.481 | 5.81E-06 | 3.80E-05 |
| g__Ileibacterium | -2.04 | 0.514 | 8.48E-05 | 4.02E-04 |
| g__Clostridia_UCG_014 | -2 | 0.527 | 1.66E-04 | 7.29E-04 |
| g__Lachnoclostridium | -1.65 | 0.439 | 1.84E-04 | 8.05E-04 |
| g__Intestinimonas | -1.69 | 0.503 | 8.64E-04 | 0.00314 |
| g__ASF356 | 1.51 | 0.486 | 0.00196 | 0.00629 |
| g__Muribaculum | -1.29 | 0.476 | 0.00679 | 0.0189 |
| g__Lachnospiraceae_FCS020_group | 1.38 | 0.509 | 0.007 | 0.0193 |
| g__Tuzzerella | -1.12 | 0.43 | 0.00925 | 0.0245 |
| g__Eubacterium_xylanophilum_group | -1.03 | 0.416 | 0.0138 | 0.0347 |
| g__Incertae_Sedis | -0.94 | 0.396 | 0.0178 | 0.0427 |
| Ileibacterium_valens | -2.04 | 0.514 | 8.48E-05 | 3.91E-04 |
| mouse_gut | 1.26 | 0.446 | 0.00486 | 0.0132 |
| uncultured_Barnesiella | -1.2 | 0.444 | 0.007 | 0.0178 |
| uncultured_Bacteroidales | 0.641 | 0.274 | 0.0197 | 0.0408 |

**Legend:** d (domain), p (phylum), c (class), o (order), f (family), g (genus), standard error (St. Error), false-discovery rate adjusted p-value (FDR)

**Table S5**. Pairwise comparisons between the facilities for Bray-Curtis index.

| **Comparisons** | **F-value** | **R-squared** | **P-value** | **FDR** |
| --- | --- | --- | --- | --- |
| Facility 1 vs Facility 3 | 3.4034 | 0.006884 | 0.009 | 0.009 |
| Facility 1 vs Facility 2 | 43.06 | 0.071999 | 0.001 | 0.0015 |
| Facility 3 vs Facility 2 | 6.4507 | 0.07293 | 0.001 | 0.0015 |

**Table S6**. Differences in gut microbiome taxa across animal house facilities relative to animal facility #1 (showing taxa with q<0.05).

| Name | Log2FC | St.Error | P-value | FDR |
| --- | --- | --- | --- | --- |
| Facility 1/Facility 3 |  |  |  |  |
| p__Deferribacterota | -5.15 | 0.886 | 1.04E-08 | 1.09E-07 |
| p__Proteobacteria | 2.48 | 0.645 | 1.32E-04 | 4.77E-04 |
| p__Bacteroidota | 0.492 | 0.159 | 0.00207 | 0.00556 |
| c__Deferribacteres | -5.15 | 0.886 | 1.04E-08 | 1.20E-07 |
| c__Gammaproteobacteria | 3.22 | 0.639 | 6.26E-07 | 5.34E-06 |
| c__Bacteroidia | 0.492 | 0.159 | 0.00207 | 0.006 |
| c__Bacilli | -1.97 | 0.791 | 0.0132 | 0.0344 |
| c__Clostridia | -1.12 | 0.451 | 0.0137 | 0.0346 |
| o__Deferribacterales | -5.15 | 0.886 | 1.04E-08 | 1.17E-07 |
| o__Burkholderiales | 3.22 | 0.639 | 6.26E-07 | 4.97E-06 |
| o__Clostridia_UCG_014 | 2.51 | 0.729 | 6.20E-04 | 0.00233 |
| o__Bacteroidales | 0.492 | 0.159 | 0.00207 | 0.00651 |
| o__Lactobacillales | -1.9 | 0.695 | 0.00643 | 0.0187 |
| o__Acholeplasmatales | -2.87 | 1.11 | 0.0101 | 0.0278 |
| o__Lachnospirales | -1.29 | 0.509 | 0.0114 | 0.0307 |
| o__Peptococcales | 1.32 | 0.521 | 0.0113 | 0.0307 |
| o__Erysipelotrichales | -1.71 | 0.689 | 0.0135 | 0.0359 |
| f__Deferribacteraceae | -5.15 | 0.886 | 1.04E-08 | 1.08E-07 |
| f__Sutterellaceae | 3.22 | 0.639 | 6.26E-07 | 4.86E-06 |
| f__Butyricicoccaceae | 2.32 | 0.534 | 1.69E-05 | 9.96E-05 |
| f__Clostridia_UCG_014 | 2.51 | 0.729 | 6.20E-04 | 0.00236 |
| f__Lactobacillaceae | -2.29 | 0.706 | 0.00125 | 0.00416 |
| f__Ruminococcaceae | -1.77 | 0.582 | 0.00254 | 0.00806 |
| f__Muribaculaceae | 1.02 | 0.367 | 0.00585 | 0.0168 |
| f__Acholeplasmataceae | -2.87 | 1.11 | 0.0101 | 0.0276 |
| f__Lachnospiraceae | -1.29 | 0.509 | 0.0114 | 0.0306 |
| f__Peptococcaceae | 1.32 | 0.521 | 0.0113 | 0.0306 |
| f__Rikenellaceae | 1.66 | 0.666 | 0.0129 | 0.0344 |
| f__Bacteroidaceae | 0.953 | 0.385 | 0.0137 | 0.0355 |
| f__Erysipelotrichaceae | -1.71 | 0.689 | 0.0135 | 0.0355 |
| g__Mucispirillum | -5.15 | 0.886 | 1.04E-08 | 1.22E-07 |
| g__Intestinimonas | 3.75 | 0.696 | 1.06E-07 | 1.02E-06 |
| g__Parasutterella | 3.22 | 0.639 | 6.26E-07 | 4.95E-06 |
| g__ASF356 | -3.27 | 0.672 | 1.55E-06 | 1.12E-05 |
| Not_Assigned | -1.82 | 0.411 | 1.15E-05 | 6.89E-05 |
| g__Roseburia | -3.38 | 0.768 | 1.29E-05 | 7.61E-05 |
| g__UCG_009 | 2.32 | 0.534 | 1.69E-05 | 9.34E-05 |
| g__Blautia | -3.06 | 0.753 | 5.40E-05 | 2.63E-04 |
| g__A2 | -2.98 | 0.749 | 7.97E-05 | 3.70E-04 |
| g__Anaerotruncus | -2.59 | 0.723 | 3.70E-04 | 0.00144 |
| g__Eubacterium_xylanophilum_group | 2.03 | 0.575 | 4.58E-04 | 0.00173 |
| g__Clostridia_UCG_014 | 2.51 | 0.729 | 6.20E-04 | 0.00224 |
| g__Lactobacillus | -2.29 | 0.706 | 0.00125 | 0.00405 |
| g__Lachnospiraceae_FCS020_group | -2.2 | 0.704 | 0.0019 | 0.00591 |
| g__uncultured | -1.41 | 0.491 | 0.00416 | 0.0123 |
| g__Lachnoclostridium | -1.73 | 0.607 | 0.00468 | 0.0135 |
| g__Muribaculaceae | 1.03 | 0.367 | 0.00511 | 0.0145 |
| g__Lachnospiraceae_UCG_001 | -2.46 | 0.915 | 0.00729 | 0.0195 |
| g__Anaeroplasma | -2.87 | 1.11 | 0.0101 | 0.0258 |
| g__Alistipes | 1.66 | 0.666 | 0.0129 | 0.0323 |
| g__Bacteroides | 0.953 | 0.385 | 0.0137 | 0.034 |
| Clostridium_leptum | -3.79 | 0.629 | 3.11E-09 | 2.93E-08 |
| uncultured_Barnesiella | 3.08 | 0.614 | 7.08E-07 | 4.25E-06 |
| Lachnospiraceae_bacterium | -3.06 | 0.753 | 5.40E-05 | 2.37E-04 |
| mouse_gut | -2.13 | 0.617 | 6.10E-04 | 0.002 |
| unidentified | -1.63 | 0.577 | 0.0048 | 0.013 |
| Facility 1/Facility 2 |  |  |  |  |
| p__Bacteroidota | 0.988 | 0.193 | 4.10E-07 | 3.04E-06 |
| p__Deferribacterota | 4.75 | 1.07 | 1.19E-05 | 5.74E-05 |
| p__Verrucomicrobiota | 3.25 | 1.1 | 0.00341 | 0.00914 |
| p__Firmicutes | -1.26 | 0.455 | 0.00575 | 0.0145 |
| c__Bacteroidia | 0.988 | 0.193 | 4.10E-07 | 3.69E-06 |
| c__Bacilli | -4.42 | 0.958 | 4.96E-06 | 3.09E-05 |
| c__Deferribacteres | 4.75 | 1.07 | 1.19E-05 | 6.40E-05 |
| c__Verrucomicrobiae | 3.25 | 1.1 | 0.00341 | 0.00952 |
| o__Lactobacillales | -4.4 | 0.842 | 2.51E-07 | 2.12E-06 |
| o__Bacteroidales | 0.988 | 0.193 | 4.10E-07 | 3.26E-06 |
| o__Erysipelotrichales | -3.77 | 0.835 | 7.92E-06 | 4.75E-05 |
| o__Deferribacterales | 4.75 | 1.07 | 1.19E-05 | 6.81E-05 |
| o__Acholeplasmatales | -4.11 | 1.35 | 0.00243 | 0.00744 |
| o__Verrucomicrobiales | 3.25 | 1.1 | 0.00341 | 0.01 |
| f__Lactobacillaceae | -4.28 | 0.855 | 7.64E-07 | 6.05E-06 |
| f__Eubacterium_coprostanoligenes_group | 3.16 | 0.67 | 3.17E-06 | 2.20E-05 |
| f__Erysipelotrichaceae | -3.77 | 0.835 | 7.92E-06 | 4.90E-05 |
| f__Deferribacteraceae | 4.75 | 1.07 | 1.19E-05 | 7.11E-05 |
| f__Muribaculaceae | 1.74 | 0.445 | 1.03E-04 | 4.73E-04 |
| f__Acholeplasmataceae | -4.11 | 1.35 | 0.00243 | 0.00769 |
| f__Akkermansiaceae | 3.25 | 1.1 | 0.00341 | 0.0104 |
| g__Muribaculum | -6.11 | 0.798 | 8.55E-14 | 1.81E-12 |
| g__ASF356 | -4.1 | 0.815 | 6.61E-07 | 5.29E-06 |
| g__Lactobacillus | -4.28 | 0.855 | 7.64E-07 | 6.05E-06 |
| g__Ileibacterium | -4.2 | 0.862 | 1.42E-06 | 1.02E-05 |
| g__Eubacterium_coprostanoligenes_group | 3.16 | 0.67 | 3.17E-06 | 2.11E-05 |
| g__Dorea | -3.11 | 0.684 | 6.72E-06 | 4.21E-05 |
| g__Lachnospiraceae_FCS020_group | -3.82 | 0.853 | 9.05E-06 | 5.46E-05 |
| g__Mucispirillum | 4.75 | 1.07 | 1.19E-05 | 6.94E-05 |
| g__Lachnospiraceae_UCG_006 | -3.34 | 0.792 | 2.87E-05 | 1.49E-04 |
| g__Muribaculaceae | 1.79 | 0.445 | 6.24E-05 | 2.92E-04 |
| g__Anaeroplasma | -4.11 | 1.35 | 0.00243 | 0.00756 |
| g__Roseburia | -2.8 | 0.931 | 0.00279 | 0.0086 |
| g__Akkermansia | 3.25 | 1.1 | 0.00341 | 0.0103 |
| g__Eubacterium_xylanophilum_group | -1.81 | 0.697 | 0.00984 | 0.0257 |
| g__Intestinimonas | -2.09 | 0.844 | 0.0134 | 0.0343 |
| uncultured_Bacteroidales | 2.26 | 0.459 | 1.19E-06 | 7.39E-06 |
| Ileibacterium_valens | -4.2 | 0.862 | 1.42E-06 | 8.49E-06 |
| mouse_gut | -2.9 | 0.747 | 1.19E-04 | 4.63E-04 |

**Legend:** d (domain), p (phylum), c (class), o (order), f (family), g (genus), standard error (St. Error), false-discovery rate adjusted p-value (FDR)

**Table S7**. Differences in gut microbiome taxa relative to young mice (showing taxa with q<0.05).

| Name | Log2FC | St.Error | P-value | FDR |
| --- | --- | --- | --- | --- |
| Young/Aged |  |  |  |  |
| p__Firmicutes | -0.992 | 0.24 | 4.18E-05 | 1.70E-04 |
| p__Deferribacterota | 2.3 | 0.567 | 5.63E-05 | 2.22E-04 |
| p__Actinobacteriota | 1.6 | 0.396 | 6.25E-05 | 2.39E-04 |
| c__Bacilli | -2.51 | 0.506 | 9.01E-07 | 7.30E-06 |
| c__Deferribacteres | 2.3 | 0.567 | 5.63E-05 | 2.53E-04 |
| c__Coriobacteriia | 1.6 | 0.396 | 6.25E-05 | 2.74E-04 |
| c__Gammaproteobacteria | 1.62 | 0.409 | 8.68E-05 | 3.70E-04 |
| o__Acholeplasmatales | -4.48 | 0.712 | 6.63E-10 | 9.42E-09 |
| o__Lactobacillales | 2.38 | 0.445 | 1.24E-07 | 1.08E-06 |
| o__Clostridia_UCG_014 | -1.89 | 0.467 | 5.74E-05 | 2.92E-04 |
| o__Deferribacterales | 2.3 | 0.567 | 5.63E-05 | 2.92E-04 |
| o__Coriobacteriales | 1.6 | 0.396 | 6.25E-05 | 3.12E-04 |
| o__Burkholderiales | 1.62 | 0.409 | 8.68E-05 | 4.18E-04 |
| o__Clostridia_vadinBB60_group | -1.35 | 0.406 | 9.02E-04 | 0.00317 |
| o__Erysipelotrichales | 1.31 | 0.441 | 0.0032 | 0.0096 |
| f__Eubacterium_coprostanoligenes_group | 2.54 | 0.354 | 2.34E-12 | 3.57E-11 |
| f__Acholeplasmataceae | -4.48 | 0.712 | 6.63E-10 | 8.75E-09 |
| f__Rikenellaceae | -2.54 | 0.426 | 4.58E-09 | 5.18E-08 |
| f__Lactobacillaceae | 2.45 | 0.452 | 8.40E-08 | 7.92E-07 |
| f__Deferribacteraceae | 2.3 | 0.567 | 5.63E-05 | 2.93E-04 |
| f__Clostridia_UCG_014 | -1.89 | 0.467 | 5.74E-05 | 2.95E-04 |
| f__Atopobiaceae | 1.6 | 0.396 | 6.25E-05 | 3.13E-04 |
| f__Sutterellaceae | 1.62 | 0.409 | 8.68E-05 | 4.19E-04 |
| f__Clostridia_vadinBB60_group | -1.35 | 0.406 | 9.02E-04 | 0.00322 |
| f__Erysipelotrichaceae | 1.31 | 0.441 | 0.0032 | 0.00982 |
| f__Bacteroidaceae | 0.698 | 0.246 | 0.00481 | 0.0141 |
| g__Muribaculum | -4.93 | 0.422 | 2.32E-28 | 1.19E-26 |
| g__Eubacterium_coprostanoligenes_group | 2.54 | 0.354 | 2.34E-12 | 4.02E-11 |
| g__Desulfovibrio | 2.59 | 0.39 | 7.79E-11 | 1.15E-09 |
| g__Anaeroplasma | -4.48 | 0.712 | 6.63E-10 | 8.84E-09 |
| g__Alistipes | -2.54 | 0.426 | 4.58E-09 | 5.78E-08 |
| g__Lactobacillus | 2.45 | 0.452 | 8.40E-08 | 8.40E-07 |
| g__ASF356 | -2.3 | 0.43 | 1.32E-07 | 1.25E-06 |
| g__Mucispirillum | 2.3 | 0.567 | 5.63E-05 | 2.72E-04 |
| g__Clostridia_UCG_014 | -1.89 | 0.467 | 5.74E-05 | 2.75E-04 |
| g__Coriobacteriaceae_UCG_002 | 1.6 | 0.396 | 6.25E-05 | 2.96E-04 |
| g__Parasutterella | 1.62 | 0.409 | 8.68E-05 | 3.95E-04 |
| g__Lachnoclostridium | 1.37 | 0.389 | 4.68E-04 | 0.00175 |
| g__Lachnospiraceae_FCS020_group | 1.55 | 0.45 | 6.08E-04 | 0.00222 |
| g__Clostridia_vadinBB60_group | -1.35 | 0.406 | 9.02E-04 | 0.00308 |
| g__Oscillibacter | -1.32 | 0.399 | 9.47E-04 | 0.0032 |
| g__Eubacterium_xylanophilum_group | 1.2 | 0.368 | 0.00116 | 0.00384 |
| g__GCA_900066575 | -1.25 | 0.42 | 0.00319 | 0.00961 |
| g__Bacteroides | 0.698 | 0.246 | 0.00481 | 0.0138 |
| g__Lachnospiraceae_UCG_001 | -1.63 | 0.586 | 0.00571 | 0.0159 |
| Not_Assigned | 0.673 | 0.263 | 0.0108 | 0.0273 |
| g__Faecalibaculum | 1.07 | 0.426 | 0.0126 | 0.0318 |
| uncultured_Barnesiella | 2.98 | 0.393 | 1.56E-13 | 2.44E-12 |
| uncultured_Bacteroidales | 1.53 | 0.243 | 5.94E-10 | 5.88E-09 |
| uncultured_bacterium | -0.66 | 0.139 | 2.52E-06 | 1.42E-05 |
| Not_Assigned | 0.432 | 0.103 | 2.93E-05 | 1.35E-04 |
| unidentified | -1.37 | 0.369 | 2.28E-04 | 8.52E-04 |
| mouse_gut | 0.998 | 0.395 | 0.0117 | 0.0264 |

**Legend:** d (domain), p (phylum), c (class), o (order), f (family), g (genus), standard error (St. Error), false-discovery rate adjusted p-value (FDR)

**Table S8**. Differences in gut microbiome taxa between sexes relative to male mice (showing taxa with q<0.05).

| Name | Log2FC | St.Error | P-value | FDR |
| --- | --- | --- | --- | --- |
| Male/female |  |  |  |  |
| p__Deferribacterota | 2.03 | 0.479 | 2.52E-05 | 1.09E-04 |
| p__Desulfobacterota | -0.889 | 0.262 | 7.39E-04 | 0.00227 |
| p__Actinobacteriota | -1.12 | 0.334 | 8.55E-04 | 0.00256 |
| c__Deferribacteres | 2.03 | 0.479 | 2.52E-05 | 1.24E-04 |
| c__Desulfovibrionia | -0.889 | 0.262 | 7.39E-04 | 0.00255 |
| c__Coriobacteriia | -1.12 | 0.334 | 8.55E-04 | 0.00283 |
| c__Alphaproteobacteria | 1.23 | 0.472 | 0.00966 | 0.0257 |
| o__Clostridia_vadinBB60_group | 1.85 | 0.342 | 9.84E-08 | 8.86E-07 |
| o__Deferribacterales | 2.03 | 0.479 | 2.52E-05 | 1.39E-04 |
| o__Desulfovibrionales | -0.889 | 0.262 | 7.39E-04 | 0.00273 |
| o__Coriobacteriales | -1.12 | 0.334 | 8.55E-04 | 0.00308 |
| o__Rhodospirillales | 1.23 | 0.472 | 0.00966 | 0.0269 |
| f__Clostridia_vadinBB60_group | 1.85 | 0.342 | 9.84E-08 | 8.86E-07 |
| f__Deferribacteraceae | 2.03 | 0.479 | 2.52E-05 | 1.41E-04 |
| f__Desulfovibrionaceae | -0.889 | 0.262 | 7.39E-04 | 0.00276 |
| f__Atopobiaceae | -1.12 | 0.334 | 8.55E-04 | 0.00309 |
| f__Eubacterium_coprostanoligenes_group | -0.849 | 0.299 | 0.00464 | 0.0137 |
| f__uncultured | 1.23 | 0.472 | 0.00966 | 0.0268 |
| f__Rikenellaceae | 0.883 | 0.36 | 0.0144 | 0.037 |
| g__Roseburia | -2.3 | 0.415 | 4.70E-08 | 4.98E-07 |
| g__Clostridia_vadinBB60_group | 1.85 | 0.342 | 9.84E-08 | 9.58E-07 |
| g__Intestinimonas | -1.95 | 0.376 | 3.18E-07 | 2.63E-06 |
| g__Mucispirillum | 2.03 | 0.479 | 2.52E-05 | 1.33E-04 |
| g__Lachnospiraceae_UCG_001 | -1.97 | 0.494 | 7.53E-05 | 3.52E-04 |
| g__Anaerotruncus | -1.49 | 0.39 | 1.48E-04 | 6.38E-04 |
| g__Bilophila | -1.07 | 0.318 | 8.49E-04 | 0.00297 |
| g__Coriobacteriaceae_UCG_002 | -1.12 | 0.334 | 8.55E-04 | 0.00297 |
| g__Incertae_Sedis | -0.961 | 0.296 | 0.00121 | 0.00399 |
| g__Lachnoclostridium | -1.03 | 0.328 | 0.00186 | 0.00581 |
| g__Eubacterium_coprostanoligenes_group | -0.849 | 0.299 | 0.00464 | 0.0135 |
| g__Blautia | -1.06 | 0.406 | 0.0093 | 0.0239 |
| g__Eubacterium_xylanophilum_group | -0.788 | 0.311 | 0.0115 | 0.029 |
| g__Alistipes | 0.883 | 0.36 | 0.0144 | 0.0356 |
| Clostridium_leptum | 1.93 | 0.34 | 2.32E-08 | 1.79E-07 |
| Lachnospiraceae_bacterium | -1.06 | 0.406 | 0.0093 | 0.0222 |

**Legend:** d (domain), p (phylum), c (class), o (order), f (family), g (genus), standard error (St. Error), false-discovery rate adjusted p-value (FDR)

**Table S9**. Pairwise comparisons between the diets for Bray-Curtis index (q<0.05).

| **Comparisons** | **F-value** | **R-squared** | **P-value** | **FDR** |
| --- | --- | --- | --- | --- |
| Normal_chow vs High_fibre | 108.68 | 0.19315 | 0.001 | 0.00125 |
| Normal_chow vs No_fibre | 108.74 | 0.18886 | 0.001 | 0.00125 |
| Normal_chow vs AING93 | 46.572 | 0.097315 | 0.001 | 0.00125 |
| Normal_chow vs HAMSAB | 7.0146 | 0.016984 | 0.001 | 0.00125 |
| High_fibre vs No_fibre | 26.283 | 0.17606 | 0.001 | 0.00125 |
| High_fibre vs AING93 | 13.707 | 0.13477 | 0.001 | 0.00125 |
| High_fibre vs HAMSAB | 4.117 | 0.062269 | 0.001 | 0.00125 |
| No_fibre vs AING93 | 23.149 | 0.18646 | 0.001 | 0.00125 |
| No_fibre vs HAMSAB | 4.496 | 0.056557 | 0.002 | 0.002222 |
| AING93 vs HAMSAB | 2.3348 | 0.055151 | 0.044 | 0.044 |

**Table S10**. Differences in gut microbiome taxa across diets relative to the control (normal chow) diet (showing taxa with q<0.05).

| Taxa | Log2FC | St.Error | P-value | FDR |
| --- | --- | --- | --- | --- |
| Normal chow/AIN93G |  |  |  |  |
| p__Deferribacterota | 3.47 | 0.752 | 4.93E-06 | 2.82E-05 |
| p__Desulfobacterota | -1.53 | 0.411 | 2.30E-04 | 8.06E-04 |
| c__Deferribacteres | 3.47 | 0.752 | 4.93E-06 | 3.07E-05 |
| c__Gammaproteobacteria | 2.12 | 0.542 | 1.02E-04 | 4.15E-04 |
| c__Desulfovibrionia | -1.53 | 0.411 | 2.30E-04 | 8.88E-04 |
| o__Deferribacterales | 3.47 | 0.752 | 4.93E-06 | 3.25E-05 |
| o__Burkholderiales | 2.12 | 0.542 | 1.02E-04 | 4.77E-04 |
| o__Desulfovibrionales | -1.53 | 0.411 | 2.30E-04 | 9.71E-04 |
| o__Erysipelotrichales | -2.16 | 0.585 | 2.51E-04 | 1.04E-03 |
| o__Clostridia_UCG_014 | 1.5 | 0.618 | 1.56E-02 | 3.93E-02 |
| f__Streptococcaceae | -2.36 | 0.406 | 1.02E-08 | 1.08E-07 |
| f__Eubacterium_coprostanoligenes_group | 2.45 | 0.469 | 2.65E-07 | 2.18E-06 |
| f__Deferribacteraceae | 3.47 | 0.752 | 4.93E-06 | 3.37E-05 |
| f__Butyricicoccaceae | 1.93 | 0.453 | 2.44E-05 | 1.38E-04 |
| f__Muribaculaceae | 1.3 | 0.311 | 3.66E-05 | 1.99E-04 |
| f__Bacteroidaceae | -1.3 | 0.327 | 8.27E-05 | 4.04E-04 |
| f__Sutterellaceae | 2.12 | 0.542 | 1.02E-04 | 4.83E-04 |
| f__Desulfovibrionaceae | -1.53 | 0.411 | 2.30E-04 | 9.80E-04 |
| f__Erysipelotrichaceae | -2.16 | 0.585 | 2.51E-04 | 1.06E-03 |
| f__Tannerellaceae | -1.55 | 0.447 | 5.55E-04 | 2.17E-03 |
| f__Clostridia_UCG_014 | 1.5 | 0.618 | 1.56E-02 | 3.90E-02 |
| g__Lactococcus | -2.36 | 0.406 | 1.02E-08 | 1.22E-07 |
| g__Bilophila | -2.88 | 0.499 | 1.29E-08 | 1.50E-07 |
| g__Roseburia | 3.52 | 0.652 | 9.49E-08 | 9.36E-07 |
| g__Dorea | 2.54 | 0.478 | 1.58E-07 | 1.46E-06 |
| g__Eubacterium_coprostanoligenes_group | 2.45 | 0.469 | 2.65E-07 | 2.24E-06 |
| g__Lachnospiraceae_UCG_006 | 2.81 | 0.554 | 5.62E-07 | 4.55E-06 |
| g__Eubacterium_xylanophilum_group | 2.31 | 0.488 | 2.89E-06 | 1.95E-05 |
| g__Mucispirillum | 3.47 | 0.752 | 4.93E-06 | 3.29E-05 |
| g__Tuzzerella | -2.21 | 0.505 | 1.42E-05 | 8.24E-05 |
| g__UCG_009 | 1.93 | 0.453 | 2.44E-05 | 1.30E-04 |
| g__Muribaculaceae | 1.31 | 0.311 | 2.91E-05 | 1.52E-04 |
| g__Faecalibaculum | -2.35 | 0.564 | 3.74E-05 | 1.92E-04 |
| g__Bacteroides | -1.3 | 0.327 | 8.27E-05 | 3.81E-04 |
| g__Parasutterella | 2.12 | 0.542 | 1.02E-04 | 4.61E-04 |
| g__Lachnospiraceae_FCS020_group | 2.18 | 0.597 | 2.84E-04 | 1.15E-03 |
| g__Lachnospiraceae_UCG_001 | 2.2 | 0.776 | 4.73E-03 | 1.36E-02 |
| g__uncultured | -1.11 | 0.416 | 8.00E-03 | 0.021 |
| g__Blautia | -1.69 | 0.638 | 0.00845 | 0.0221 |
| g__Clostridia_UCG_014 | 1.5 | 0.618 | 0.0156 | 0.0375 |
| Clostridium_leptum | 3.44 | 0.533 | 2.38E-10 | 2.62E-09 |
| Lactococcus_lactis | -2.36 | 0.406 | 1.02E-08 | 8.45E-08 |
| uncultured_Bacteroidales | 1.65 | 0.321 | 3.85E-07 | 2.38E-06 |
| uncultured_Barnesiella | 2.29 | 0.521 | 1.36E-05 | 6.73E-05 |
| Lachnospiraceae_bacterium | -1.69 | 0.638 | 8.45E-03 | 2.04E-02 |
| Normal chow/HAMSAB |  |  |  |  |
| o__Peptococcales | 2.14 | 0.71 | 2.73E-03 | 8.66E-03 |
| f__Peptococcaceae | 2.14 | 0.71 | 2.73E-03 | 9.55E-03 |
| f__Muribaculaceae | 1.19 | 0.5 | 1.73E-02 | 4.54E-02 |
| g__Bilophila | -2.5 | 0.801 | 1.89E-03 | 6.32E-03 |
| g__Blautia | -2.96 | 1.02 | 4.07E-03 | 1.28E-02 |
| g__Anaerotruncus | -2.52 | 0.984 | 1.07E-02 | 2.89E-02 |
| g__GCA_900066575 | -2.27 | 0.894 | 1.15E-02 | 3.06E-02 |
| g__Muribaculaceae | 1.23 | 0.5 | 1.45E-02 | 3.75E-02 |
| g__Dorea | 1.82 | 0.768 | 1.83E-02 | 4.51E-02 |
| uncultured_Barnesiella | 3.77 | 0.836 | 7.80E-06 | 4.54E-05 |
| uncultured_Bacteroidales | 2.06 | 0.516 | 7.68E-05 | 3.54E-04 |
| Lachnospiraceae_bacterium | -2.96 | 1.02 | 4.07E-03 | 0.0129 |
| Normal chow/High-fibre |  |  |  |  |
| p__Verrucomicrobiota | -5.65 | 0.83 | 2.72E-11 | 3.11E-10 |
| p__Deferribacterota | 3.7 | 0.808 | 5.62E-06 | 2.90E-05 |
| p__Bacteroidota | -0.567 | 0.145 | 1.03E-04 | 3.61E-04 |
| p__Proteobacteria | -1.53 | 0.588 | 9.29E-03 | 2.30E-02 |
| c__Verrucomicrobiae | -5.65 | 0.83 | 2.72E-11 | 3.67E-10 |
| c__Deferribacteres | 3.7 | 0.808 | 5.62E-06 | 3.21E-05 |
| c__Bacteroidia | -0.567 | 0.145 | 1.03E-04 | 4.08E-04 |
| o__Verrucomicrobiales | -5.65 | 0.83 | 2.72E-11 | 4.08E-10 |
| o__Acholeplasmatales | 4.68 | 1.01 | 4.84E-06 | 3.18E-05 |
| o__Deferribacterales | 3.7 | 0.808 | 5.62E-06 | 3.53E-05 |
| o__Bacteroidales | -0.567 | 0.145 | 1.03E-04 | 4.57E-04 |
| o__Clostridia_vadinBB60_group | 1.82 | 0.578 | 0.00174 | 0.00546 |
| o__Peptococcales | -1.49 | 0.475 | 0.00177 | 0.00549 |
| o__Oscillospirales | 1.12 | 0.484 | 0.0213 | 0.0472 |
| f__Bacteroidaceae | -3.08 | 0.351 | 2.30E-17 | 5.35E-16 |
| f__Butyricicoccaceae | 3.71 | 0.487 | 1.14E-13 | 1.96E-12 |
| f__Akkermansiaceae | -5.65 | 0.83 | 2.72E-11 | 3.36E-10 |
| f__Eubacterium_coprostanoligenes_group | 2.96 | 0.504 | 7.19E-09 | 6.78E-08 |
| f__Muribaculaceae | 1.89 | 0.335 | 2.62E-08 | 2.20E-07 |
| f__Acholeplasmataceae | 4.68 | 1.01 | 4.84E-06 | 3.04E-05 |
| f__Deferribacteraceae | 3.7 | 0.808 | 5.62E-06 | 3.35E-05 |
| f__Tannerellaceae | -2.03 | 0.481 | 2.81E-05 | 1.39E-04 |
| f__Lactobacillaceae | 2.37 | 0.643 | 2.47E-04 | 9.99E-04 |
| f__Rikenellaceae | 2.11 | 0.607 | 5.66E-04 | 0.00215 |
| f__Clostridia_vadinBB60_group | 1.82 | 0.578 | 0.00174 | 0.00547 |
| f__Peptococcaceae | -1.49 | 0.475 | 0.00177 | 0.00552 |
| f__Oscillospiraceae | 1.16 | 0.487 | 0.0171 | 0.0394 |
| g__Bacteroides | -3.08 | 0.351 | 2.30E-17 | 6.12E-16 |
| g__Roseburia | 5.47 | 0.7 | 2.94E-14 | 6.22E-13 |
| g__UCG_009 | 3.71 | 0.487 | 1.14E-13 | 2.11E-12 |
| g__Lachnospiraceae_UCG_006 | 4.33 | 0.596 | 1.27E-12 | 1.95E-11 |
| g__A2 | 4.83 | 0.683 | 4.66E-12 | 6.72E-11 |
| g__Eubacterium_xylanophilum_group | 3.67 | 0.524 | 7.38E-12 | 1.02E-10 |
| g__Akkermansia | -5.65 | 0.83 | 2.72E-11 | 3.48E-10 |
| g__Dorea | 3.47 | 0.514 | 3.66E-11 | 4.48E-10 |
| g__Eubacterium_coprostanoligenes_group | 2.96 | 0.504 | 7.19E-09 | 7.19E-08 |
| g__Muribaculaceae | 1.88 | 0.334 | 2.99E-08 | 2.76E-07 |
| g__Desulfovibrio | 3.03 | 0.555 | 7.06E-08 | 6.05E-07 |
| g__Lachnospiraceae_FCS020_group | 3.24 | 0.641 | 6.11E-07 | 4.35E-06 |
| g__Anaeroplasma | 4.68 | 1.01 | 4.84E-06 | 2.93E-05 |
| g__Mucispirillum | 3.7 | 0.808 | 5.62E-06 | 3.32E-05 |
| g__Tuzzerella | 2.46 | 0.543 | 6.83E-06 | 3.90E-05 |
| g__Lachnospiraceae_UCG_001 | 3.57 | 0.834 | 2.15E-05 | 1.07E-04 |
| g__Intestinimonas | 2.6 | 0.635 | 4.85E-05 | 2.24E-04 |
| g__Lactobacillus | 2.37 | 0.643 | 2.47E-04 | 9.73E-04 |
| g__Oscillibacter | 1.97 | 0.567 | 5.46E-04 | 0.0019 |
| g__Alistipes | 2.11 | 0.607 | 5.66E-04 | 0.00195 |
| g__Faecalibaculum | -1.95 | 0.606 | 0.00138 | 0.00408 |
| g__Lachnoclostridium | 1.75 | 0.554 | 0.0017 | 0.00494 |
| g__Clostridia_vadinBB60_group | 1.82 | 0.578 | 0.00174 | 0.00503 |
| g__Incertae_Sedis | 1.42 | 0.499 | 0.00448 | 0.012 |
| g__Dubosiella | 0.994 | 0.425 | 0.0197 | 0.0424 |
| uncultured_Bacteroidales | 4.57 | 0.345 | 6.36E-35 | 3.15E-33 |
| Clostridium_leptum | 5.66 | 0.573 | 2.73E-21 | 6.01E-20 |
| Not_Assigned | -0.737 | 0.146 | 6.22E-07 | 3.62E-06 |
| unidentified | 2.17 | 0.526 | 4.35E-05 | 1.83E-04 |
| mouse_gut | 1.77 | 0.562 | 0.0017 | 0.00475 |
| uncultured_bacterium | 0.594 | 0.197 | 0.00275 | 0.00736 |
| uncultured_Barnesiella | 1.67 | 0.56 | 0.00292 | 0.00771 |
| Dubosiella_newyorkensis | 0.994 | 0.425 | 0.0197 | 0.0395 |
| Normal chow/low-fibre |  |  |  |  |
| p__Desulfobacterota | -3.93 | 0.455 | 5.79E-17 | 1.22E-15 |
| p__Deferribacterota | 5.74 | 0.831 | 1.40E-11 | 1.60E-10 |
| p__Verrucomicrobiota | -5.63 | 0.854 | 1.02E-10 | 1.07E-09 |
| p__Actinobacteriota | -2.53 | 0.58 | 1.53E-05 | 6.43E-05 |
| p__Bacteroidota | -0.519 | 0.149 | 5.42E-04 | 0.00159 |
| p__Firmicutes | 1.04 | 0.352 | 0.00323 | 0.00754 |
| c__Desulfovibrionia | -3.93 | 0.455 | 5.79E-17 | 1.56E-15 |
| c__Deferribacteres | 5.74 | 0.831 | 1.40E-11 | 1.89E-10 |
| c__Verrucomicrobiae | -5.63 | 0.854 | 1.02E-10 | 1.27E-09 |
| c__Coriobacteriia | -2.53 | 0.58 | 1.53E-05 | 7.30E-05 |
| c__Clostridia | 1.52 | 0.423 | 3.57E-04 | 0.0012 |
| c__Bacteroidia | -0.519 | 0.149 | 5.42E-04 | 0.00169 |
| c__Gammaproteobacteria | 1.74 | 0.599 | 0.0039 | 0.00987 |
| o__Desulfovibrionales | -3.93 | 0.455 | 5.79E-17 | 1.56E-15 |
| o__Deferribacterales | 5.74 | 0.831 | 1.40E-11 | 2.10E-10 |
| o__Verrucomicrobiales | -5.63 | 0.854 | 1.02E-10 | 1.37E-09 |
| o__Lachnospirales | 2.43 | 0.477 | 4.86E-07 | 3.36E-06 |
| o__Clostridia_vadinBB60_group | 2.98 | 0.594 | 7.35E-07 | 4.84E-06 |
| o__Coriobacteriales | -2.53 | 0.58 | 1.53E-05 | 7.95E-05 |
| o__Clostridia_UCG_014 | 2.8 | 0.683 | 4.94E-05 | 2.30E-04 |
| o__Bacteroidales | -0.519 | 0.149 | 5.42E-04 | 0.00181 |
| o__Lactobacillales | -2.26 | 0.652 | 5.52E-04 | 0.00182 |
| o__Erysipelotrichales | -2.12 | 0.647 | 0.00112 | 0.00338 |
| o__Burkholderiales | 1.74 | 0.599 | 0.0039 | 0.0101 |
| o__Acholeplasmatales | 2.77 | 1.04 | 0.00828 | 0.0205 |
| o__Peptococcales | -1.14 | 0.489 | 0.0206 | 0.0438 |
| f__Tannerellaceae | -4.46 | 0.494 | 3.00E-18 | 7.43E-17 |
| f__Desulfovibrionaceae | -3.93 | 0.455 | 5.79E-17 | 1.27E-15 |
| f__Bacteroidaceae | -2.55 | 0.361 | 4.89E-12 | 6.24E-11 |
| f__Deferribacteraceae | 5.74 | 0.831 | 1.40E-11 | 1.73E-10 |
| f__Akkermansiaceae | -5.63 | 0.854 | 1.02E-10 | 1.22E-09 |
| f__Streptococcaceae | -2.88 | 0.449 | 2.76E-10 | 3.22E-09 |
| f__Lachnospiraceae | 2.43 | 0.477 | 4.86E-07 | 3.38E-06 |
| f__Clostridia_vadinBB60_group | 2.98 | 0.594 | 7.35E-07 | 4.93E-06 |
| f__Eubacterium_coprostanoligenes_group | 2.58 | 0.519 | 8.90E-07 | 5.88E-06 |
| f__Atopobiaceae | -2.53 | 0.58 | 1.53E-05 | 7.98E-05 |
| f__Clostridia_UCG_014 | 2.8 | 0.683 | 4.94E-05 | 2.35E-04 |
| f__Erysipelotrichaceae | -2.12 | 0.647 | 0.00112 | 0.00371 |
| f__Muribaculaceae | 1.12 | 0.344 | 0.00127 | 0.00399 |
| f__Sutterellaceae | 1.74 | 0.599 | 0.0039 | 0.011 |
| f__Acholeplasmataceae | 2.77 | 1.04 | 0.00828 | 0.0216 |
| f__Peptococcaceae | -1.14 | 0.489 | 0.0206 | 0.0464 |
| g__Bilophila | -5.95 | 0.552 | 9.87E-25 | 3.74E-23 |
| g__Roseburia | 6.13 | 0.72 | 1.65E-16 | 4.10E-15 |
| g__Lachnospiraceae_NK4A136_group | 4.89 | 0.58 | 2.83E-16 | 6.57E-15 |
| g__Lachnospiraceae_FCS020_group | 5.34 | 0.66 | 3.60E-15 | 7.63E-14 |
| g__Bacteroides | -2.55 | 0.361 | 4.89E-12 | 7.33E-11 |
| g__Mucispirillum | 5.74 | 0.831 | 1.40E-11 | 2.01E-10 |
| g__A2 | 4.67 | 0.703 | 7.41E-11 | 9.70E-10 |
| g__Akkermansia | -5.63 | 0.854 | 1.02E-10 | 1.26E-09 |
| g__Faecalibaculum | -4.03 | 0.624 | 2.29E-10 | 2.80E-09 |
| g__Lactococcus | -2.88 | 0.449 | 2.76E-10 | 3.30E-09 |
| g__Lachnospiraceae_UCG_001 | 4.91 | 0.858 | 1.75E-08 | 1.64E-07 |
| g__Desulfovibrio | 2.93 | 0.571 | 3.94E-07 | 2.90E-06 |
| g__Dorea | 2.67 | 0.529 | 5.87E-07 | 4.22E-06 |
| g__Clostridia_vadinBB60_group | 2.98 | 0.594 | 7.35E-07 | 5.14E-06 |
| g__Eubacterium_coprostanoligenes_group | 2.58 | 0.519 | 8.90E-07 | 6.05E-06 |
| g__Coriobacteriaceae_UCG_002 | -2.53 | 0.58 | 1.53E-05 | 7.96E-05 |
| g__Dubosiella | -1.91 | 0.437 | 1.54E-05 | 7.96E-05 |
| g__Clostridia_UCG_014 | 2.8 | 0.683 | 4.94E-05 | 2.26E-04 |
| g__Eubacterium_xylanophilum_group | 2.16 | 0.539 | 6.91E-05 | 3.02E-04 |
| g__Tuzzerella | -1.86 | 0.558 | 8.98E-04 | 2.92E-03 |
| g__Muribaculaceae | 1.1 | 0.344 | 1.43E-03 | 4.35E-03 |
| g__Parasutterella | 1.74 | 0.599 | 3.90E-03 | 1.08E-02 |
| g__Anaeroplasma | 2.77 | 1.04 | 8.28E-03 | 2.08E-02 |
| g__ASF356 | 1.65 | 0.63 | 9.06E-03 | 2.25E-02 |
| Not_Assigned | -0.88 | 0.385 | 2.29E-02 | 4.97E-02 |
| Clostridium_leptum | 5.58 | 0.59 | 9.26E-20 | 2.29E-18 |
| uncultured_Barnesiella | 5.08 | 0.576 | 1.49E-17 | 3.22E-16 |
| mouse_gut | 4.03 | 0.578 | 9.61E-12 | 9.52E-11 |
| Lactococcus_lactis | -2.88 | 0.449 | 2.76E-10 | 2.49E-09 |
| uncultured_Bacteroidales | 1.56 | 0.355 | 1.35E-05 | 6.23E-05 |
| Dubosiella_newyorkensis | -1.91 | 0.437 | 1.54E-05 | 6.76E-05 |
| Not_Assigned | -0.588 | 0.15 | 1.02E-04 | 3.66E-04 |
| unidentified | 2.02 | 0.541 | 2.03E-04 | 6.80E-04 |

**Legend:** d (domain), p (phylum), c (class), o (order), f (family), g (genus), standard error (St. Error), false-discovery rate adjusted p-value (FDR)

**Table S11**. Differences in gut microbiome taxa between the compartments relative to the large intestine (showing taxa with q<0.05).

| Name | Log2FC | St.Error | P-value | FDR |
| --- | --- | --- | --- | --- |
| Large intestine/small intestine |  |  |  |  |
| p__Firmicutes | 4.06 | 0.217 | 1.35E-60 | 1.70E-58 |
| p__Deferribacterota | 4.27 | 0.514 | 7.07E-16 | 1.48E-14 |
| p__Bacteroidota | -0.448 | 0.0922 | 1.57E-06 | 9.90E-06 |
| p__Proteobacteria | 1.56 | 0.374 | 3.66E-05 | 1.54E-04 |
| c__Clostridia | 5.27 | 0.262 | 4.91E-68 | 7.96E-66 |
| c__Deferribacteres | 4.27 | 0.514 | 7.07E-16 | 1.91E-14 |
| c__Alphaproteobacteria | 3.11 | 0.506 | 1.65E-09 | 2.23E-08 |
| c__Bacteroidia | -0.448 | 0.0922 | 1.57E-06 | 1.11E-05 |
| o__Clostridia_vadinBB60_group | 7.9 | 0.367 | 8.79E-75 | 2.37E-72 |
| o__Oscillospirales | 6.05 | 0.308 | 1.82E-65 | 2.46E-63 |
| o__Lachnospirales | 5.05 | 0.295 | 5.91E-53 | 5.32E-51 |
| o__Clostridia_UCG_014 | 5.33 | 0.422 | 3.02E-32 | 2.04E-30 |
| o__Deferribacterales | 4.27 | 0.514 | 7.07E-16 | 1.91E-14 |
| o__Peptococcales | 2.3 | 0.302 | 1.33E-13 | 3.00E-12 |
| o__Rhodospirillales | 3.11 | 0.506 | 1.65E-09 | 2.13E-08 |
| o__Bacteroidales | -0.448 | 0.0922 | 1.57E-06 | 1.09E-05 |
| o__Lactobacillales | -1.55 | 0.403 | 1.36E-04 | 5.92E-04 |
| o__Acholeplasmatales | 2.06 | 0.645 | 0.00148 | 0.00483 |
| f__Bacteroidaceae | 6.06 | 0.223 | 2.07E-103 | 8.19E-101 |
| f__Clostridia_vadinBB60_group | 7.9 | 0.367 | 8.79E-75 | 1.74E-72 |
| f__Oscillospiraceae | 5.49 | 0.31 | 5.83E-56 | 7.70E-54 |
| f__Lachnospiraceae | 5.05 | 0.295 | 5.91E-53 | 5.85E-51 |
| f__Tannerellaceae | 4.89 | 0.306 | 1.34E-47 | 1.06E-45 |
| f__Ruminococcaceae | 5.13 | 0.337 | 8.47E-44 | 5.59E-42 |
| f__Rikenellaceae | 5.29 | 0.386 | 5.97E-37 | 3.38E-35 |
| f__Clostridia_UCG_014 | 5.33 | 0.422 | 3.02E-32 | 1.49E-30 |
| f__Eubacterium_coprostanoligenes_group | 3.6 | 0.321 | 1.88E-26 | 5.74E-25 |
| f__Deferribacteraceae | 4.27 | 0.514 | 7.07E-16 | 1.65E-14 |
| f__Butyricicoccaceae | 2.41 | 0.309 | 3.17E-14 | 6.76E-13 |
| f__Peptococcaceae | 2.3 | 0.302 | 1.33E-13 | 2.40E-12 |
| f__uncultured | 3.11 | 0.506 | 1.65E-09 | 2.05E-08 |
| f__Lactobacillaceae | -1.58 | 0.409 | 1.19E-04 | 5.50E-04 |
| f__Muribaculaceae | -0.736 | 0.213 | 5.85E-04 | 0.00227 |
| f__Acholeplasmataceae | 2.06 | 0.645 | 0.00148 | 0.0049 |
| g__Bacteroides | 6.06 | 0.223 | 2.07E-103 | 1.49E-100 |
| Not_Assigned | 6.08 | 0.238 | 2.33E-95 | 8.38E-93 |
| g__Clostridia_vadinBB60_group | 7.9 | 0.367 | 8.79E-75 | 2.11E-72 |
| g__uncultured | 5.04 | 0.285 | 7.02E-56 | 1.26E-53 |
| g__Alistipes | 5.29 | 0.386 | 5.97E-37 | 7.17E-35 |
| g__Oscillibacter | 4.69 | 0.361 | 7.92E-34 | 8.14E-32 |
| g__Clostridia_UCG_014 | 5.33 | 0.422 | 3.02E-32 | 2.72E-30 |
| g__Colidextribacter | 4.34 | 0.354 | 1.07E-30 | 8.59E-29 |
| g__Lachnospiraceae_NK4A136_group | 4.38 | 0.358 | 1.63E-30 | 1.17E-28 |
| g__Incertae_Sedis | 3.85 | 0.317 | 3.09E-30 | 2.03E-28 |
| g__Eubacterium_xylanophilum_group | 3.96 | 0.333 | 4.21E-29 | 2.53E-27 |
| g__Lachnoclostridium | 3.98 | 0.352 | 9.95E-27 | 4.22E-25 |
| g__Eubacterium_coprostanoligenes_group | 3.6 | 0.321 | 1.88E-26 | 7.53E-25 |
| g__Intestinimonas | 3.76 | 0.404 | 2.97E-19 | 9.71E-18 |
| g__Lachnospiraceae_FCS020_group | 3.51 | 0.408 | 8.09E-17 | 2.24E-15 |
| g__Bilophila | 2.89 | 0.341 | 1.98E-16 | 5.09E-15 |
| g__Mucispirillum | 4.27 | 0.514 | 7.07E-16 | 1.70E-14 |
| g__A2 | 3.42 | 0.434 | 1.97E-14 | 4.57E-13 |
| g__UCG_009 | 2.41 | 0.309 | 3.17E-14 | 7.08E-13 |
| g__GCA_900066575 | 2.67 | 0.381 | 7.12E-12 | 1.19E-10 |
| g__Lachnospiraceae_UCG_006 | 2.6 | 0.379 | 1.87E-11 | 3.07E-10 |
| g__Roseburia | 2.83 | 0.445 | 4.14E-10 | 5.62E-09 |
| g__Dorea | 1.61 | 0.327 | 1.16E-06 | 8.60E-06 |
| g__Anaerotruncus | 1.85 | 0.419 | 1.27E-05 | 7.58E-05 |
| g__Lachnospiraceae_UCG_001 | 2.06 | 0.53 | 1.14E-04 | 5.05E-04 |
| g__Lactobacillus | -1.58 | 0.409 | 1.19E-04 | 5.24E-04 |
| g__Tuzzerella | 1.27 | 0.345 | 2.55E-04 | 0.00107 |
| g__Muribaculaceae | -0.74 | 0.213 | 5.46E-04 | 0.00201 |
| g__Anaeroplasma | 2.06 | 0.645 | 0.00148 | 0.00475 |
| g__Faecalibaculum | -1.07 | 0.386 | 0.00573 | 0.0159 |
| g__Blautia | 1.13 | 0.436 | 0.0101 | 0.0258 |
| unidentified | 5.05 | 0.334 | 2.90E-43 | 2.87E-41 |
| Not_Assigned | 0.931 | 0.0929 | 7.61E-22 | 2.15E-20 |
| mouse_gut | 2.7 | 0.357 | 1.92E-13 | 2.72E-12 |
| uncultured_Barnesiella | -1.73 | 0.356 | 1.46E-06 | 8.49E-06 |
| Clostridium_leptum | 1.34 | 0.364 | 2.72E-04 | 9.96E-04 |
| Lachnospiraceae_bacterium | 1.13 | 0.436 | 0.0101 | 0.0239 |
| uncultured_Bacteroidales | -0.561 | 0.22 | 0.011 | 0.0252 |

**Legend:** d (domain), p (phylum), c (class), o (order), f (family), g (genus), standard error (St. Error), false-discovery rate adjusted p-value (FDR)

**Table S12**. Pairwise comparisons between the sequencing batches for Bray-Curtis index.

| **Pair** | **F-value** | **R-squared** | **P-value** | **FDR** |
| --- | --- | --- | --- | --- |
| May-21 vs Aug-21 | 8.6092 | 0.02644 | 0.001 | 0.001 |
| May-21 vs Nov-22 | 28.1154 | 0.202101 | 0.001 | 0.001 |
| May-21 vs Oct-20 | 13.1709 | 0.122896 | 0.001 | 0.001 |
| May-21 vs Dec-21 | 27.3616 | 0.200655 | 0.001 | 0.001 |
| May-21 vs Mar-20 | 36.8297 | 0.185233 | 0.001 | 0.001 |
| Aug-21 vs Nov-22 | 58.6442 | 0.158222 | 0.001 | 0.001 |
| Aug-21 vs Oct-20 | 27.5621 | 0.085447 | 0.001 | 0.001 |
| Aug-21 vs Dec-21 | 58.2847 | 0.15826 | 0.001 | 0.001 |
| Aug-21 vs Mar-20 | 15.4778 | 0.040895 | 0.001 | 0.001 |
| Nov-22 vs Oct-20 | 17.6923 | 0.165825 | 0.001 | 0.001 |
| Nov-22 vs Dec-21 | 23.9105 | 0.186932 | 0.001 | 0.001 |
| Nov-22 vs Mar-20 | 110.034 | 0.41206 | 0.001 | 0.001 |
| Oct-20 vs Dec-21 | 8.6798 | 0.090717 | 0.001 | 0.001 |
| Oct-20 vs Mar-20 | 56.8606 | 0.288837 | 0.001 | 0.001 |
| Dec-21 vs Mar-20 | 104.79 | 0.403363 | 0.001 | 0.001 |

**Table S13**. Differences in gut microbiome taxa between sequencing batches relative to Aug-21 batch (showing taxa with q<0.05).

| **Taxa** | **Log2FC** | **St. Error** | **P-value** | **FDR** |
| --- | --- | --- | --- | --- |
| Aug21/Dec21 |  |  |  |  |
| p__Verrucomicrobiota | 7.83 | 0.876 | 6.06E-18 | 1.91E-16 |
| p__Deferribacterota | -6.14 | 0.852 | 1.99E-12 | 2.50E-11 |
| p__Bacteroidota | 0.833 | 0.153 | 7.68E-08 | 5.09E-07 |
| p__Actinobacteriota | -2.55 | 0.595 | 2.20E-05 | 8.40E-05 |
| p__Desulfobacterota | -1.83 | 0.466 | 9.45E-05 | 3.05E-04 |
| p__Firmicutes | -1.17 | 0.361 | 1.27E-03 | 0.00292 |
| c__Verrucomicrobiae | 7.83 | 0.876 | 6.06E-18 | 2.46E-16 |
| c__Deferribacteres | -6.14 | 0.852 | 1.99E-12 | 3.22E-11 |
| c__Bacteroidia | 0.833 | 0.153 | 7.68E-08 | 6.22E-07 |
| c__Coriobacteriia | -2.55 | 0.595 | 2.20E-05 | 9.63E-05 |
| c__Desulfovibrionia | -1.83 | 0.466 | 9.45E-05 | 3.64E-04 |
| c__Bacilli | -2.65 | 0.76 | 5.22E-04 | 0.00163 |
| c__Clostridia | -1.43 | 0.434 | 1.03E-03 | 2.82E-03 |
| c__Gammaproteobacteria | -1.48 | 0.614 | 1.62E-02 | 3.54E-02 |
| o__Verrucomicrobiales | 7.83 | 0.876 | 6.06E-18 | 2.34E-16 |
| o__Deferribacterales | -6.14 | 0.852 | 1.99E-12 | 3.83E-11 |
| o__Erysipelotrichales | -4.12 | 0.663 | 1.01E-09 | 1.43E-08 |
| o__Clostridia_UCG_014 | 3.97 | 0.701 | 2.36E-08 | 2.45E-07 |
| o__Bacteroidales | 0.833 | 0.153 | 7.68E-08 | 6.91E-07 |
| o__Coriobacteriales | -2.55 | 0.595 | 2.20E-05 | 1.10E-04 |
| o__Desulfovibrionales | -1.83 | 0.466 | 9.45E-05 | 4.11E-04 |
| o__Lachnospirales | -1.88 | 0.489 | 1.33E-04 | 5.48E-04 |
| o__Oscillospirales | -1.76 | 0.511 | 6.10E-04 | 2.11E-03 |
| o__Lactobacillales | -2.02 | 0.668 | 0.00267 | 0.00721 |
| o__Burkholderiales | -1.48 | 0.614 | 0.0162 | 0.0363 |
| f__Akkermansiaceae | 7.83 | 0.876 | 6.06E-18 | 1.85E-16 |
| f__Eubacterium_coprostanoligenes_group | -4.07 | 0.532 | 8.87E-14 | 1.85E-12 |
| f__Deferribacteraceae | -6.14 | 0.852 | 1.99E-12 | 3.15E-11 |
| f__Streptococcaceae | -2.93 | 0.46 | 4.13E-10 | 5.28E-09 |
| f__Erysipelotrichaceae | -4.12 | 0.663 | 1.01E-09 | 1.21E-08 |
| f__Clostridia_UCG_014 | 3.97 | 0.701 | 2.36E-08 | 2.28E-07 |
| f__Atopobiaceae | -2.55 | 0.595 | 2.20E-05 | 1.15E-04 |
| f__Desulfovibrionaceae | -1.83 | 0.466 | 9.45E-05 | 4.25E-04 |
| f__Lachnospiraceae | -1.88 | 0.489 | 1.33E-04 | 5.73E-04 |
| f__Oscillospiraceae | -1.95 | 0.514 | 1.69E-04 | 6.90E-04 |
| f__Ruminococcaceae | -1.66 | 0.56 | 3.15E-03 | 9.16E-03 |
| f__Butyricicoccaceae | -1.32 | 0.513 | 1.01E-02 | 0.0261 |
| f__Sutterellaceae | -1.48 | 0.614 | 0.0162 | 0.039 |
| f__Muribaculaceae | 0.847 | 0.353 | 0.0167 | 0.0399 |
| g__Akkermansia | 7.83 | 0.876 | 6.06E-18 | 1.98E-16 |
| g__Blautia | -6.38 | 0.724 | 1.63E-17 | 5.09E-16 |
| g__Eubacterium_coprostanoligenes_group | -4.07 | 0.532 | 8.87E-14 | 1.93E-12 |
| g__Mucispirillum | -6.14 | 0.852 | 1.99E-12 | 3.67E-11 |
| g__Lactococcus | -2.93 | 0.46 | 4.13E-10 | 5.73E-09 |
| g__Clostridia_UCG_014 | 3.97 | 0.701 | 2.36E-08 | 2.70E-07 |
| g__Anaerotruncus | -3.84 | 0.695 | 5.04E-08 | 5.26E-07 |
| g__Desulfovibrio | -3.09 | 0.585 | 1.87E-07 | 1.68E-06 |
| g__Tuzzerella | -2.74 | 0.572 | 2.13E-06 | 1.48E-05 |
| g__Faecalibaculum | -2.86 | 0.639 | 9.10E-06 | 5.60E-05 |
| g__Coriobacteriaceae_UCG_002 | -2.55 | 0.595 | 2.20E-05 | 1.23E-04 |
| g__Colidextribacter | -2.44 | 0.588 | 3.80E-05 | 1.97E-04 |
| g__Lachnospiraceae_UCG_006 | -2.57 | 0.628 | 4.84E-05 | 2.45E-04 |
| g__Lachnospiraceae_FCS020_group | -2.72 | 0.677 | 6.61E-05 | 3.13E-04 |
| g__ASF356 | -2.53 | 0.646 | 1.00E-04 | 4.55E-04 |
| g__Dubosiella | -1.74 | 0.448 | 1.12E-04 | 4.98E-04 |
| g__Bilophila | -2.13 | 0.565 | 1.87E-04 | 7.57E-04 |
| g__Ileibacterium | -2.49 | 0.684 | 2.89E-04 | 0.0011 |
| g__Muribaculum | -2.3 | 0.633 | 3.05E-04 | 0.00115 |
| g__Oscillibacter | -2.07 | 0.598 | 5.97E-04 | 0.00208 |
| g__uncultured | -1.62 | 0.472 | 6.63E-04 | 0.00226 |
| g__Lachnoclostridium | -1.97 | 0.584 | 8.11E-04 | 0.00269 |
| g__Roseburia | -2.38 | 0.739 | 0.00132 | 0.00407 |
| Not_Assigned | -1.21 | 0.395 | 0.00232 | 0.00686 |
| g__Incertae_Sedis | -1.53 | 0.526 | 0.00384 | 0.0109 |
| g__A2 | -1.98 | 0.72 | 0.00631 | 0.0162 |
| g__UCG_009 | -1.32 | 0.513 | 0.0101 | 0.0243 |
| g__Muribaculaceae | 0.86 | 0.353 | 0.0151 | 0.0344 |
| g__Parasutterella | -1.48 | 0.614 | 0.0162 | 0.0364 |
| Lachnospiraceae_bacterium | -6.38 | 0.724 | 1.63E-17 | 5.37E-16 |
| Lactococcus_lactis | -2.93 | 0.46 | 4.13E-10 | 5.12E-09 |
| Clostridium_leptum | -3.4 | 0.605 | 2.86E-08 | 2.36E-07 |
| mouse_gut | -3.05 | 0.593 | 3.73E-07 | 2.73E-06 |
| Dubosiella_newyorkensis | -1.74 | 0.448 | 1.12E-04 | 4.62E-04 |
| Ileibacterium_valens | -2.49 | 0.684 | 2.89E-04 | 0.00105 |
| uncultured_Barnesiella | 1.62 | 0.59 | 0.00627 | 0.0155 |
| uncultured_bacterium | -0.531 | 0.208 | 0.0111 | 0.0247 |
| Not_Assigned | 0.386 | 0.154 | 0.0126 | 0.0271 |
| unidentified | -1.3 | 0.555 | 0.0198 | 0.0408 |
| Aug21/Mar20 |  |  |  |  |
| p__Actinobacteriota | -1.74 | 0.285 | 2.03E-09 | 2.46E-08 |
| p__Verrucomicrobiota | -1.93 | 0.42 | 5.44E-06 | 3.11E-05 |
| p__Bacteroidota | -0.171 | 0.0733 | 0.0202 | 0.0479 |
| c__Coriobacteriia | -1.74 | 0.285 | 2.03E-09 | 2.90E-08 |
| c__Verrucomicrobiae | -1.93 | 0.42 | 5.44E-06 | 3.39E-05 |
| c__Gammaproteobacteria | -1.15 | 0.294 | 1.13E-04 | 4.95E-04 |
| c__Bacilli | -1 | 0.364 | 0.00624 | 0.0177 |
| c__Bacteroidia | -0.171 | 0.0733 | 0.0202 | 0.0487 |
| o__Erysipelotrichales | -2.27 | 0.318 | 2.99E-12 | 6.22E-11 |
| o__Lactobacillales | -2.13 | 0.32 | 7.18E-11 | 1.38E-09 |
| o__Coriobacteriales | -1.74 | 0.285 | 2.03E-09 | 2.88E-08 |
| o__Peptococcales | -1.4 | 0.24 | 8.70E-09 | 1.07E-07 |
| o__Verrucomicrobiales | -1.93 | 0.42 | 5.44E-06 | 3.86E-05 |
| o__Burkholderiales | -1.15 | 0.294 | 1.13E-04 | 5.54E-04 |
| o__Acholeplasmatales | 1.24 | 0.513 | 0.0161 | 0.0396 |
| o__Bacteroidales | -0.171 | 0.0733 | 0.0202 | 0.0473 |
| f__Streptococcaceae | -2.61 | 0.221 | 6.61E-29 | 2.91E-27 |
| f__Erysipelotrichaceae | -2.27 | 0.318 | 2.99E-12 | 5.15E-11 |
| f__Atopobiaceae | -1.74 | 0.285 | 2.03E-09 | 2.87E-08 |
| f__Peptococcaceae | -1.4 | 0.24 | 8.70E-09 | 1.08E-07 |
| f__Akkermansiaceae | -1.93 | 0.42 | 5.44E-06 | 3.78E-05 |
| f__Lactobacillaceae | -1.49 | 0.325 | 5.74E-06 | 3.86E-05 |
| f__Sutterellaceae | -1.15 | 0.294 | 1.13E-04 | 5.66E-04 |
| f__Acholeplasmataceae | 1.24 | 0.513 | 0.0161 | 0.0405 |
| g__Lactococcus | -2.61 | 0.221 | 6.61E-29 | 3.66E-27 |
| g__Dubosiella | -2.31 | 0.215 | 1.56E-24 | 6.25E-23 |
| g__Coriobacteriaceae_UCG_002 | -1.74 | 0.285 | 2.03E-09 | 3.11E-08 |
| g__Ileibacterium | -1.94 | 0.328 | 6.13E-09 | 8.33E-08 |
| g__Faecalibaculum | -1.68 | 0.307 | 6.06E-08 | 6.92E-07 |
| g__Akkermansia | -1.93 | 0.42 | 5.44E-06 | 3.76E-05 |
| g__Lactobacillus | -1.49 | 0.325 | 5.74E-06 | 3.90E-05 |
| g__Blautia | -1.51 | 0.347 | 1.58E-05 | 9.63E-05 |
| g__Parasutterella | -1.15 | 0.294 | 1.13E-04 | 5.53E-04 |
| g__Bilophila | -0.955 | 0.271 | 4.64E-04 | 0.00184 |
| g__Lachnoclostridium | -0.833 | 0.28 | 0.00306 | 0.00984 |
| g__Intestinimonas | -0.936 | 0.321 | 0.00369 | 0.0116 |
| g__A2 | -0.925 | 0.345 | 0.00764 | 0.0208 |
| g__Desulfovibrio | -0.734 | 0.281 | 0.00911 | 0.024 |
| g__Anaerotruncus | -0.813 | 0.333 | 0.0151 | 0.0372 |
| g__Anaeroplasma | 1.24 | 0.513 | 0.0161 | 0.0389 |
| g__Eubacterium_xylanophilum_group | -0.629 | 0.265 | 0.0181 | 0.0432 |
| g__Lachnospiraceae_UCG_006 | -0.709 | 0.301 | 0.0189 | 0.0451 |
| Lactococcus_lactis | -2.61 | 0.221 | 6.61E-29 | 3.27E-27 |
| Dubosiella_newyorkensis | -2.31 | 0.215 | 1.56E-24 | 6.19E-23 |
| Ileibacterium_valens | -1.94 | 0.328 | 6.13E-09 | 6.07E-08 |
| Lachnospiraceae_bacterium | -1.51 | 0.347 | 1.58E-05 | 7.62E-05 |
| Not_Assigned | 0.291 | 0.0739 | 9.16E-05 | 3.51E-04 |
| mouse_gut | -1.12 | 0.284 | 9.62E-05 | 3.59E-04 |
| Clostridium_leptum | 0.798 | 0.29 | 0.0061 | 0.0157 |
| uncultured_bacterium | -0.247 | 0.0999 | 0.0138 | 0.0307 |
| Aug21/May21 |  |  |  |  |
| p__Desulfobacterota | -1.27 | 0.294 | 1.94E-05 | 1.02E-04 |
| p__Proteobacteria | -1.11 | 0.39 | 0.00472 | 0.0129 |
| p__Deferribacterota | -1.32 | 0.537 | 0.0145 | 0.0365 |
| c__Desulfovibrionia | -1.27 | 0.294 | 1.94E-05 | 1.12E-04 |
| c__Alphaproteobacteria | -1.72 | 0.529 | 0.00119 | 0.00409 |
| c__Deferribacteres | -1.32 | 0.537 | 0.0145 | 0.0378 |
| o__Desulfovibrionales | -1.27 | 0.294 | 1.94E-05 | 1.38E-04 |
| o__Erysipelotrichales | -1.68 | 0.417 | 6.75E-05 | 4.05E-04 |
| o__Rhodospirillales | -1.72 | 0.529 | 0.00119 | 0.00452 |
| o__Oscillospirales | -0.87 | 0.322 | 0.00708 | 0.0215 |
| o__Deferribacterales | -1.32 | 0.537 | 0.0145 | 0.0386 |
| f__Streptococcaceae | -1.33 | 0.29 | 5.09E-06 | 4.03E-05 |
| f__Desulfovibrionaceae | -1.27 | 0.294 | 1.94E-05 | 1.37E-04 |
| f__Erysipelotrichaceae | -1.68 | 0.417 | 6.75E-05 | 4.11E-04 |
| f__Tannerellaceae | -1.09 | 0.319 | 6.58E-04 | 0.00272 |
| f__uncultured | -1.72 | 0.529 | 0.00119 | 0.00444 |
| f__Bacteroidaceae | -0.699 | 0.233 | 0.00283 | 0.0092 |
| f__Ruminococcaceae | -0.991 | 0.352 | 0.00512 | 0.0155 |
| f__Oscillospiraceae | -0.905 | 0.324 | 0.00534 | 0.016 |
| f__Deferribacteraceae | -1.32 | 0.537 | 0.0145 | 0.038 |
| g__Bilophila | -1.89 | 0.356 | 1.63E-07 | 1.78E-06 |
| g__Lactococcus | -1.33 | 0.29 | 5.09E-06 | 3.67E-05 |
| g__Colidextribacter | -1.51 | 0.37 | 4.90E-05 | 2.80E-04 |
| g__Dorea | -1.22 | 0.341 | 3.99E-04 | 0.00167 |
| Not_Assigned | -0.829 | 0.249 | 9.27E-04 | 0.00341 |
| g__Incertae_Sedis | -1.08 | 0.331 | 0.00115 | 0.00409 |
| g__Ileibacterium | -1.37 | 0.431 | 0.00158 | 0.00535 |
| g__uncultured | -0.915 | 0.297 | 0.00218 | 0.00708 |
| g__Bacteroides | -0.699 | 0.233 | 0.00283 | 0.009 |
| g__Mucispirillum | -1.32 | 0.537 | 0.0145 | 0.0372 |
| g__Blautia | -1.08 | 0.456 | 0.0177 | 0.0442 |
| Lactococcus_lactis | -1.33 | 0.29 | 5.09E-06 | 3.15E-05 |
| Ileibacterium_valens | -1.37 | 0.431 | 0.00158 | 0.00512 |
| Clostridium_leptum | -0.954 | 0.381 | 0.0125 | 0.0288 |
| Lachnospiraceae_bacterium | -1.08 | 0.456 | 0.0177 | 0.0402 |
| Aug21/Nov22 |  |  |  |  |
| p__Actinobacteriota | -5.25 | 0.542 | 1.35E-20 | 5.69E-19 |
| p__Bacteroidota | 1.3 | 0.139 | 2.77E-19 | 8.73E-18 |
| p__Firmicutes | -1.82 | 0.329 | 4.99E-08 | 2.99E-07 |
| p__Desulfobacterota | -2.3 | 0.425 | 9.02E-08 | 5.17E-07 |
| p__Proteobacteria | -1.45 | 0.565 | 0.0107 | 0.0237 |
| c__Coriobacteriia | -5.25 | 0.542 | 1.35E-20 | 7.32E-19 |
| c__Bacteroidia | 1.3 | 0.139 | 2.77E-19 | 1.12E-17 |
| c__Bacilli | -5.19 | 0.692 | 2.53E-13 | 3.72E-12 |
| c__Desulfovibrionia | -2.3 | 0.425 | 9.02E-08 | 6.09E-07 |
| c__Clostridia | -1.67 | 0.395 | 2.88E-05 | 1.11E-04 |
| c__Gammaproteobacteria | -2.32 | 0.559 | 3.82E-05 | 1.44E-04 |
| o__Erysipelotrichales | -7.23 | 0.604 | 1.73E-29 | 9.33E-28 |
| o__Coriobacteriales | -5.25 | 0.542 | 1.35E-20 | 5.23E-19 |
| o__Bacteroidales | 1.3 | 0.139 | 2.77E-19 | 9.35E-18 |
| o__Lactobacillales | -4.46 | 0.608 | 8.33E-13 | 1.18E-11 |
| o__Desulfovibrionales | -2.3 | 0.425 | 9.02E-08 | 6.96E-07 |
| o__Oscillospirales | -2.32 | 0.466 | 8.65E-07 | 5.31E-06 |
| o__Lachnospirales | -2.19 | 0.446 | 1.21E-06 | 7.13E-06 |
| o__Burkholderiales | -2.32 | 0.559 | 3.82E-05 | 1.69E-04 |
| o__Acholeplasmatales | -3.17 | 0.975 | 0.00123 | 0.00357 |
| f__Erysipelotrichaceae | -7.23 | 0.604 | 1.73E-29 | 7.61E-28 |
| f__Streptococcaceae | -4.84 | 0.419 | 7.96E-28 | 2.87E-26 |
| f__Atopobiaceae | -5.25 | 0.542 | 1.35E-20 | 4.13E-19 |
| f__Ruminococcaceae | -3.09 | 0.51 | 2.47E-09 | 2.65E-08 |
| f__Desulfovibrionaceae | -2.3 | 0.425 | 9.02E-08 | 7.94E-07 |
| f__Lachnospiraceae | -2.19 | 0.446 | 1.21E-06 | 7.52E-06 |
| f__Oscillospiraceae | -2.08 | 0.468 | 1.09E-05 | 5.92E-05 |
| f__Lactobacillaceae | -2.71 | 0.618 | 1.42E-05 | 7.50E-05 |
| f__Sutterellaceae | -2.32 | 0.559 | 3.82E-05 | 1.82E-04 |
| f__Butyricicoccaceae | -1.89 | 0.468 | 5.91E-05 | 2.66E-04 |
| f__Eubacterium_coprostanoligenes_group | -1.62 | 0.484 | 8.58E-04 | 0.00278 |
| f__Bacteroidaceae | 1.1 | 0.337 | 0.00116 | 0.00362 |
| f__Acholeplasmataceae | -3.17 | 0.975 | 0.00123 | 0.00377 |
| f__Muribaculaceae | 0.795 | 0.321 | 0.0137 | 0.0324 |
| g__Ileibacterium | -9.85 | 0.623 | 1.04E-46 | 1.49E-44 |
| g__Lactococcus | -4.84 | 0.419 | 7.96E-28 | 3.18E-26 |
| g__Coriobacteriaceae_UCG_002 | -5.25 | 0.542 | 1.35E-20 | 4.24E-19 |
| g__Blautia | -4.88 | 0.659 | 5.08E-13 | 8.92E-12 |
| g__Incertae_Sedis | -3.51 | 0.479 | 8.65E-13 | 1.48E-11 |
| g__Desulfovibrio | -3.64 | 0.533 | 2.32E-11 | 3.33E-10 |
| Not_Assigned | -2.45 | 0.36 | 2.76E-11 | 3.89E-10 |
| g__Anaerotruncus | -4.28 | 0.633 | 3.67E-11 | 5.09E-10 |
| g__Faecalibaculum | -3.73 | 0.583 | 3.23E-10 | 4.15E-09 |
| g__Dubosiella | -2.39 | 0.408 | 8.46E-09 | 9.51E-08 |
| g__Lachnoclostridium | -3.05 | 0.532 | 1.58E-08 | 1.67E-07 |
| g__Intestinimonas | -3.25 | 0.61 | 1.46E-07 | 1.21E-06 |
| g__Lachnospiraceae_FCS020_group | -3.24 | 0.616 | 2.17E-07 | 1.74E-06 |
| g__Colidextribacter | -2.61 | 0.535 | 1.41E-06 | 8.59E-06 |
| g__Oscillibacter | -2.45 | 0.545 | 8.65E-06 | 4.35E-05 |
| g__Lachnospiraceae_UCG_001 | -3.57 | 0.801 | 1.01E-05 | 4.99E-05 |
| g__ASF356 | -2.61 | 0.589 | 1.09E-05 | 5.33E-05 |
| g__Lactobacillus | -2.71 | 0.618 | 1.42E-05 | 6.72E-05 |
| g__GCA_900066575 | -2.51 | 0.575 | 1.49E-05 | 6.99E-05 |
| g__uncultured | -1.86 | 0.43 | 1.86E-05 | 8.49E-05 |
| g__Dorea | -2.12 | 0.494 | 2.03E-05 | 9.19E-05 |
| g__Parasutterella | -2.32 | 0.559 | 3.82E-05 | 1.60E-04 |
| g__UCG_009 | -1.89 | 0.468 | 5.91E-05 | 2.39E-04 |
| g__Lachnospiraceae_UCG_006 | -2.11 | 0.573 | 2.58E-04 | 8.97E-04 |
| g__Tuzzerella | -1.91 | 0.521 | 2.74E-04 | 9.40E-04 |
| g__Bilophila | -1.88 | 0.515 | 2.84E-04 | 9.66E-04 |
| g__Eubacterium_coprostanoligenes_group | -1.62 | 0.484 | 8.58E-04 | 0.0026 |
| g__Bacteroides | 1.1 | 0.337 | 0.00116 | 0.00335 |
| g__Anaeroplasma | -3.17 | 0.975 | 0.00123 | 0.0035 |
| g__Roseburia | -2.13 | 0.673 | 0.00167 | 0.00457 |
| g__A2 | -1.72 | 0.656 | 0.00892 | 0.0202 |
| g__Muribaculaceae | 0.78 | 0.321 | 0.0156 | 0.0332 |
| g__Lachnospiraceae_NK4A136_group | -1.3 | 0.542 | 0.0167 | 0.0354 |
| Aug21/Oct20 |  |  |  |  |
| p__Firmicutes | 1 | 0.322 | 0.0019 | 0.00557 |
| c__Clostridia | 1.2 | 0.387 | 0.00205 | 0.00659 |
| o__Oscillospirales | 1.38 | 0.456 | 0.00262 | 0.00864 |
| o__Lachnospirales | 1.06 | 0.436 | 0.0154 | 0.0404 |
| f__Muribaculaceae | 0.916 | 0.315 | 0.00376 | 0.0124 |
| f__Oscillospiraceae | 1.23 | 0.458 | 0.00729 | 0.022 |
| f__Ruminococcaceae | 1.31 | 0.499 | 0.00916 | 0.0269 |
| f__Lachnospiraceae | 1.06 | 0.436 | 0.0154 | 0.0407 |
| g__uncultured | 1.42 | 0.421 | 7.72E-04 | 0.00314 |
| g__Muribaculaceae | 0.928 | 0.315 | 0.00332 | 0.0114 |
| uncultured_Bacteroidales | 1.18 | 0.325 | 2.93E-04 | 0.0011 |
| uncultured_bacterium | 0.584 | 0.186 | 0.00175 | 0.00578 |

**Legend:** d (domain), p (phylum), c (class), o (order), f (family), g (genus), standard error (St. Error), false-discovery rate adjusted p-value (FDR)

**Table S14.** The full, shared, and unique contribution of each experimental factor to the variations in the gut microbiota composition. FDR-adjusted p-values <0.02 were considered significant. Full contribution represents how much all the experimental factors in our study explain the variations observed in the gut microbiome. Shared contribution represents how much each factor explains the variation without correcting for the other variables. Unique contribution is how much each factor explains the variations observed, after correcting for all other variables.

|  | **Factor** | **Element** | ***P* value** | **Adj.r.squared** | **Percentage of variance (%)** | **Adj. *P* value** |
| --- | --- | --- | --- | --- | --- | --- |
| 1 | full | full | NA | 0.488 | 48.800 | NA |
|  | | | | | | |
| 1 | Diet | shared | 0.001 | 0.233 | 23.300 | 0.001 |
| 2 | Sequencing batch | shared | 0.001 | 0.2091 | 20.910 | 0.001 |
| 3 | Compartment | shared | 0.001 | 0.128 | 12.800 | 0.001 |
| 4 | Animal Facility | shared | 0.001 | 0.089 | 8.900 | 0.001 |
| 5 | Genotype | shared | 0.001 | 0.051 | 5.100 | 0.001 |
| 6 | Age | shared | 0.001 | 0.047 | 4.700 | 0.001 |
| 7 | Angiotensin II treatment | shared | 0.001 | 0.028 | 2.800 | 0.001 |
| 8 | Sex | shared | 0.001 | 0.014 | 1.400 | 0.001 |
|  | | | | | | |
| 1 | Compartment | unique | 0.001 | 0.068 | 6.800 | 0.001 |
| 2 | Diet | unique | 0.001 | 0.060 | 6.000 | 0.001 |
| 3 | Sequencing batch | unique | 0.001 | 0.047 | 4.7 | 0.001 |
| 4 | Genotype | unique | 0.001 | 0.029 | 2.900 | 0.001 |
| 5 | Animal Facility | unique | 0.001 | 0.023 | 2.300 | 0.001 |
| 6 | Age | unique | 0.001 | 0.020 | 2.000 | 0.001 |
| 7 | Sex | unique | 0.001 | 0.004 | 0.400 | 0.001 |
| 8 | Angiotensin II treatment | unique | 0.001 | 0.004 | 0.400 | 0.001 |

**Legend**: Adj, adjusted; NA, not applicable.

**References**

1. Turnbaugh PJ, Bäckhed F, Fulton L, Gordon JI. Diet-Induced Obesity Is Linked to Marked but Reversible Alterations in the Mouse Distal Gut Microbiome. *Cell Host & Microbe* 2008;**3**:213-223.

2. Hufeldt MR, Nielsen DS, Vogensen FK, Midtvedt T, Hansen AK. Variation in the gut microbiota of laboratory mice is related to both genetic and environmental factors. *Comp Med* 2010;**60**:336-347.

3. Hildebrand F, Nguyen TLA, Brinkman B, Yunta RG, Cauwe B, Vandenabeele P, Liston A, Raes J. Inflammation-associated enterotypes, host genotype, cage and inter-individual effects drive gut microbiota variation in common laboratory mice. *Genome Biology* 2013;**14**:R4.

4. Rausch P, Basic M, Batra A, Bischoff SC, Blaut M, Clavel T, Gläsner J, Gopalakrishnan S, Grassl GA, Günther C, Haller D, Hirose M, Ibrahim S, Loh G, Mattner J, Nagel S, Pabst O, Schmidt F, Siegmund B, Strowig T, Volynets V, Wirtz S, Zeissig S, Zeissig Y, Bleich A, Baines JF. Analysis of factors contributing to variation in the C57BL/6J fecal microbiota across German animal facilities. *Int J Med Microbiol* 2016;**306**:343-355.

5. Hilbert T, Steinhagen F, Senzig S, Cramer N, Bekeredjian-Ding I, Parcina M, Baumgarten G, Hoeft A, Frede S, Boehm O, Klaschik S. Vendor effects on murine gut microbiota influence experimental abdominal sepsis. *J Surg Res* 2017;**211**:126-136.

6. Ericsson AC, Gagliardi J, Bouhan D, Spollen WG, Givan SA, Franklin CL. The influence of caging, bedding, and diet on the composition of the microbiota in different regions of the mouse gut. *Sci Rep* 2018;**8**:4065.

7. Montonye DR, Ericsson AC, Busi SB, Lutz C, Wardwell K, Franklin CL. Acclimation and Institutionalization of the Mouse Microbiota Following Transportation. *Frontiers in Microbiology* 2018;**9**.

8. Robertson SJ, Lemire P, Maughan H, Goethel A, Turpin W, Bedrani L, Guttman DS, Croitoru K, Girardin SE, Philpott DJ. Comparison of Co-housing and Littermate Methods for Microbiota Standardization in Mouse Models. *Cell Rep* 2019;**27**:1910-1919.e1912.

9. Wolff NS, Jacobs MC, Haak BW, Roelofs JJTH, de Vos AF, Hugenholtz F, Wiersinga WJ. Vendor effects on murine gut microbiota and its influence on lipopolysaccharide-induced lung inflammation and Gram-negative pneumonia. *Intensive Care Medicine Experimental* 2020;**8**:47.

10. Singh G, Brass A, Cruickshank SM, Knight CG. Cage and maternal effects on the bacterial communities of the murine gut. *Scientific Reports* 2021;**11**:9841.

11. Guo J, Song C, Liu Y, Wu X, Dong W, Zhu H, Xiang Z, Qin C. Characteristics of gut microbiota in representative mice strains: Implications for biological research. *Animal Model Exp Med* 2022;**5**:337-349.
